# Evaluation of Stress-Tolerant *Serratia* and *Enterobacter* as PGPR for Nutrient Solubilization and Dose-Dependent Bioformulation to Enhance Tomato Seedlings

**DOI:** 10.3390/plants14142154

**Published:** 2025-07-13

**Authors:** Indu Bhardwaj, Vijay Kumar, Somvir Singh, Arti Jamwal Sharma, Shikha Kumari, Nidhi Bhardwaj, Kanika Dulta, Lukas Peter, Richa Verma, Nitesh Kumar, Yogesh K. Ahlawat, Anurag Malik, Mohammad K. Okla, Rosa Porcel, José M. Mulet, Karthikeyan Jayabalan

**Affiliations:** 1 Division of Microbiology, Career Point University, Hamirpur 176041, Himachal Pradesh, Indiashikha.micro@cpuh.edu.in (S.K.); 2 Department of MLT, Abhilashi University, Chailchowk, Mandi 175028, Himachal Pradesh, India; 3 Department of Biotechnology, Chandigarh University, Mohali 140413, Punjab, India; vijay.micro@cpuh.edu.in; 4 Department of Biosciences, University Institute of Biotechnology, Chandigarh University, Gharuan, Mohali 140413, Punjab, India; 5 Department of Bio Science, Career Point University, Hamirpur 176041, Himachal Pradesh, India; 6 Center of Advanced Innovation Technologies, VŠB-Technical University of Ostrava, 708 00 Ostrava-Poruba, Czech Republickanikadulta10@gmail.com (K.D.); lukas.peter@vsb.cz (L.P.); 7 Department of Chemistry, Faculty of Science, University of Hradec Kralove, Rokitanskeho 62, 500 03 Hradec Kralove, Czech Republic; 8 Department of Biosciences, Himachal Pradesh University, Summer Hill, Shimla 171005, Himachal Pradesh, India; niteshchauhan7@gmail.com; 9 Department of Biotechnology, University Centre for Research and Development, Chandigarh University, Mohali 140413, Punjab, India; 10 Allied Health Sciences, Datta Meghe Institute of Higher Education and Research, Wardha 442107, Maharashtra, India; 11 Centre for Research Impact & Outcome, Chitkara University Institute of Engineering and Technology, Chitkara University, Rajpura 140401, Punjab, India; 12 Division of Research and Innovation, Uttaranchal University, Dehradun 24800, Uttarakhand, India; 13 Botany and Microbiology Department, College of Science, King Saud University, P.O. Box 2455, Riyadh 11451, Saudi Arabia; malokla@ksu.edu.sa; 14 Instituto de Biología Molecular y Celular de Plantas (IBMCP), Universitat Politècnica de València-Consejo Superior de Investigaciones Científicas, 46022 Valencia, Spain; 15 Department of Chemistry, Sathyabama Institute of Science and Technology, Chennai 600119, Tamil Nadu, India; karthikeyan.chemistry@sathyabama.ac.in

**Keywords:** dose-dependent, environmental stress, germination, IAA, PGPR, *Serratia*, *Enterobacter*

## Abstract

Plant growth-promoting rhizobacteria (PGPR) are eco-friendly and sustainable options for agrochemicals, particularly for enhancing crop productivity under stress conditions. The present research aims to isolate and characterize native PGPR from tomato rhizospheric soil and to evaluate their effectiveness as a dose-dependent response to enhance the growth of tomato seedlings. Out of 112 isolates, 10 bacterial strains were selected based on key PGPR traits, including indole-3-acetic acid (IAA), ammonia production, hydrogen cyanide (HCN), exopolysaccharide (EPS) synthesis, hydrolytic enzyme activity, potassium solubilization, antifungal activity against *Fusarium oxysporum*, and tolerance to pH and heat stress. Molecular identification via 16S rRNA gene sequencing confirmed that these isolates belong to the genera *Serratia* and *Enterobacter*. *S. marcescens* So-1 and *Enterobacter* sp. So-12 produced the highest levels of IAA (2.6–24.1 µg/mL). In vitro tomato seed germination tests using bacterial suspensions at three concentrations (10^6^, 10^7^, and 10^8^ CFU/mL) showed dose-dependent improvements, with T1 increasing germination up to 108.3% compared to the control. In polyhouse trials using cocopeat formulations, seedling growth improved noticeably. T2 increased the root length (28.3 ± 2.98 cm) by over 1560%, and the shoot length (35.7 ± 0.57 cm) increased by 55% against the control, whose root length is 1.7 ± 0.47. The chlorophyll amount of the treated leaves further showed significant results over the control. Collectively, these findings suggest that using native PGPR in a dose-dependent way can help tomato seedlings grow better and promote more sustainable crop production.

## 1. Introduction

In the 21st century, where the global agriculture system is facing various challenges, primarily driven by climate change, environmental stress, overpopulation, and other issues like these, there are further increases in the overall stress on natural resources such as water, soil, and biodiversity. These stress factors play a massive role in the decline in crop production and a heavy decline in agricultural ecosystems, which are generally considered crucial to ensure long-term food security and sustainable food production [1]. The study by the United Nations Forecasts has predicted that the global population is projected to exceed 9.7 billion by the year 2050, and the potential peak might be nearing 10.4 billion by the mid-2080s, which shows a 35% increase from 2023 levels. This swift growth in population, in addition to changing climate patterns, has further increased the challenges in food security, nutrient availability, and healthy and sustainable agriculture practices [2].

To deal with this heavy decline in crop productivity and rising environmental stress, farmers are actively adapting to the use of synthetic fertilizers and chemical fungicides to increase the sustainability of their overall yield. While these methods provide various short-term benefits, in the long run, they have led to nutrient leaching, soil degradation, water pollution, and resistant fungal strains. Using nitrogen and phosphorus-based fertilizers builds up ecological imbalances in addition to chemical fungicides, which usually contain heavy metals and organophosphorus compounds, increasing overall environmental toxicity [3,4]. The use of these substances significantly impacts beneficial soil microorganisms, affects microbial biodiversity, and hinders the essential soil functions [5]. With so many problems, the requirement of sustainable alternatives that do not affect the integrity of the ecosystem while helping to maintain productivity is essential.

Keeping that in mind, tomato (*Solanum lycopersicum*), which is a crucial crop, epitomizes the challenges faced by modern agriculture [6,7]. It is the second-highest non-starchy vegetable cultivated globally, right after potato [8]. It is an essential part of the human diet, as it provides various crucial nutrients, such as vitamin A and C, minerals, and bioactive compounds, including flavonoids, lycopene, polyphenols, and phytosterols. These compounds provide multiple health benefits, increase the fruit’s sensory qualities, and extend its shelf life [7,9].

The farming of tomatoes usually prospers in warm, frost-free climates where the optimal temperature ranges between 20 and 24 °C. In addition, a well-drained sandy loam soil with a pH of 6–7 is best suited for its cultivation. It is cultivated in a very diverse agro-climatic region worldwide, with China being the highest production hub, followed by India, which is the second-largest producer of tomato globally, with key tomato-growing states that include Andhra Pradesh, Madhya Pradesh, Karnataka, and Tamil Nadu [10,11].

In the lower Western Himalayan region of Himachal Pradesh, tomato cultivation plays a crucial role in local diets, nutrition, and rural income. Tomatoes contribute nearly one-third of the state’s total vegetable production. Solan, which is also referred to as the “City of Red Gold” [12], contributes to 46% of the overall tomato production in Himachal Pradesh, followed by Sirmaur, which produces over 30%. Other tomato-producing areas include Hamirpur, Una, Bilaspur, and the lower parts of Mandi and Kangra [13,14]. According to a report by the National Horticulture Board [15], Himachal Pradesh contributed about 2.49% of India’s total tomato production. Tomato output peaked at 577,000 metric tons in 2022 [16], but it was reported to have dropped to 474,338 metric tons in 2024, marking a 17.8% decline in total yield. This decline in the total yield of tomatoes ultimately reflects the challenges driven by unsustainable agriculture practices, which include a decrease in overall soil fertility, an imbalance in nutrients, and heavy reliance on chemical fertilizers, which, on a larger scale, affects the plant vigor and the health of the soil [17,18].

Beyond these issues, crops are heavily endangered by both biotic and abiotic stresses. *Fusarium* wilt is the most critical crop threat, caused by the soil-borne fungus *F. oxysporum* f. sp. lycopersici [19]. This pathogen invades the vascular system of the tomato plant, which causes symptoms like wilting, chlorosis, and the eventual death of the plant [18,19]. This pathogen remains persistent in the soil over an extended period, which makes it very hard to manage. While chemical fungicides are used to deal with these pathogens, these chemical fungicides only offer temporary control and very often contribute to the long-term contamination of the environment, leading to pathogens acquiring resistance to these fungicides [20]. Abiotic factors like erratic rainfall patterns and a rise in overall temperatures weaken the immunity of the plant, which leads to the vulnerability of the plant and a drop in overall productivity at the same time [21].

Despite these local challenges, a change in the global context is also observed to be changing. According to a study by the Food and Agriculture Organization (FAO) using the Autoregressive Integrated Moving Average (ARIMA) model, a sharp decrease in the annual growth rate of 9.41% between 1961 and 2022 to just 0.75% between 2023 and 2027 was observed in global tomato production. The United States, which is one of the top tomato-producing countries, is also notably expected to experience a 7.38% decline in production during this period [22]. This decline in the production of tomatoes, both locally and globally, highlights the urgent need for sustainable agricultural practices that can both ensure the long-term viability of tomato cultivation and tackle the effects of climate change.

PGPR can be considered one of the promising solutions to tackle this problem. They are beneficial microorganisms that inhabit the rhizosphere [23], which is the narrow zone of the soil surrounding plant roots. The rhizosphere acts as a dynamic interface between plant roots and soil microbes, in which PGPR exerts a range of growth-enhancing functions [24]. These bacteria act on the development of plants by fixing atmospheric nitrogen and solubilizing phosphorus and potassium. In addition to that, they help in the production of phytohormones such as indole-3-acetic acid, gibberellins, and cytokinin [25]. They produce secondary metabolites like hydrogen cyanide, lytic enzymes, and siderophores, which provide biocontrol benefits. Some of these benefits are suppressing the development of phytopathogens by restricting nutrient availability and preventing pathogen activities in the soil. PGPR also helps develop abiotic stress tolerance in plants, such as drought, high and low temperatures, and low soil pH, by improving the ability to take up water, inducing the antioxidant defense mechanisms, and regulating stress-responsive genes [26,27]. These characteristics are of great utility in open-field production systems, where the environment is difficult to manage [28,29].

Various studies have shown the positive effects of PGPR on tomato crops, including factors like better germination, growth, yield, fruit quality, and disease resistance [30]. Bacteria from the genera *Bacillus*, *Azospirillum*, *Pseudomonas*, *Rhizobium*, *Acinetobacter*, *Serratia*, and *Enterobacter* have been widely documented for their efficacy in promoting plant growth and their ability to enhance soil fertility. Out of all these genera, *Serratia* and *Enterobacter* have recently gained attention as very promising PGPR because of their dual roles in nutrient cycling and disease suppression, contributing to enhanced soil fertility and tomato productivity [30,31].

Although it has these advantages, commercial PGPR often shows inconsistent patterns in its field performance. This is primarily because non-native strains often struggle to survive, colonize, and function effectively under varied soil and climatic conditions [32]. Take the example of non-native PGPR, which tends to colonize taproots, while the native microbes typically prefer to colonize lateral roots, establishing spatial mismatches that may limit microbial performance. Additionally, introduced strains tend to compete or interfere with native soil microbiota, thus reducing the efficiency of the inoculant [33].

To minimize these limitations, bioformulation based on native PGPR has shown greater adaptability and consistent results under local conditions. Carriers such as cocopeat are generally used to deliver these formulations. Cocopeat is a biodegradable, high-retention organic medium that supports microbial survival and root colonization [34]. These bioformulations, when applied to seeds, roots, or soil, can significantly improve germination rates, increase resistance to pathogens like *F. oxysporum*, and enhance crop yield while reducing dependence on chemical inputs.

Considering these benefits, the optimization of native PGPR strains is very crucial for region-specific sustainable agriculture. This is the first study to assess the performance of native PGPR on tomato in the Hamirpur district of Himachal Pradesh through in vivo polyhouse pot trials. The PGPR strains were isolated from rhizosphere soils collected from the Lower Western Himalayan Zone. These isolates were then tested at varying concentrations (10^6^, 10^7^, and 10^8^ CFU/mL) to evaluate their influence on germination and seedling vigor. A cocopeat-based bioformulation was then developed using the most effective in vitro concentration. This study also aims to record the impacts of *Serratia* and *Enterobacter* spp. by working individually and in consortium, which offers novel insights into their potential to enhance tomato growth and disease resistance under polyhouse conditions. By taking advantage of native microbial diversity and context-specific application strategies, this study contributes a novel and regionally tailored solution to the sustainable production of tomatoes in Himachal Pradesh and globally. Although the results demonstrate strong potential under controlled conditions, further trials are required to validate their broader applicability.

## 2. Results

### 2.1. Isolation and Biochemical Characterization of Rhizobacteria

Thirty-six soil samples were collected from the tomato rhizosphere in the Lower Western Himalayan Zone of Himachal Pradesh, India. From these, 112 distinct rhizobacterial isolates were obtained through serial dilution and plating on Pikovskaya’s (PVK) agar medium. Ten potential isolates were selected and investigated in the present study. Preliminary characterization was carried out based on morphological and biochemical characteristics. Among the selected bacteria, 70% were Gram-negative bacteria. Biochemical characterization showed that 80% of the isolates were catalase positive, 90% were oxidase positive, 40% fermented mannitol, and 80% utilized citrate. Urease activity was found in 30%, while 10% were triple sugar iron (TSI) positive, and 60% were methyl red (MR) positive (Figure S1 and Table S2).

### 2.2. Characterization of Isolates for Their PGP Attributes

The isolates were distinguished on a selective basis, like their ability to produce phytohormones, solubilize macronutrients, produce beneficial metabolites, and secrete hydrolytic enzymes that support plant growth and enhance soil fertility (Figure S1).

**Figure 1:**
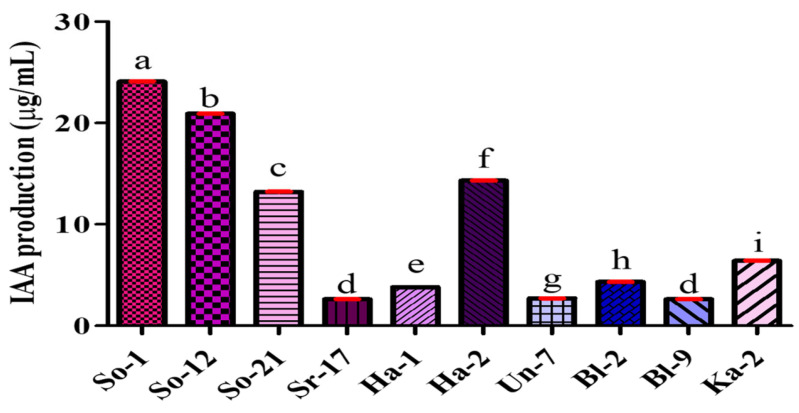
Bacterial isolates from tomato rhizosphere samples produced IAA (µg/mL). IAA production was measured in bacterial culture supernatant using Salkowski reagents. Data represents the means ± SD of at least three replicates. Statistical significance was assessed using ANOVA at a 5% significance level (*p* value < 0.05). Different lowercase letters above the bars indicate significant variation, while the same letters show non-significant differences between isolates using the Bonferroni post hoc test at α = 0.05.

Among these traits, all selected bacterial isolates produce IAA, with concentrations ranging from 2.6 to 24.1 µg/mL. IAA is a key phytohormone that functions as an effective signaling molecule in regulating plants’ growth and development processes. In consideration of preliminary IAA production screening, isolate So-1 exhibited the highest level of IAA (24.1 µg/mL), while isolates Sr-17 and Bl-9 produced the lowest concentration (2.6 µg/mL). IAA concentrations were quantified using a standard curve prepared from known concentrations of pure IAA (Figure S2). Additional details on the IAA screening of bacterial isolates are presented (Figure 1 and Table S3).

**Figure 2:**
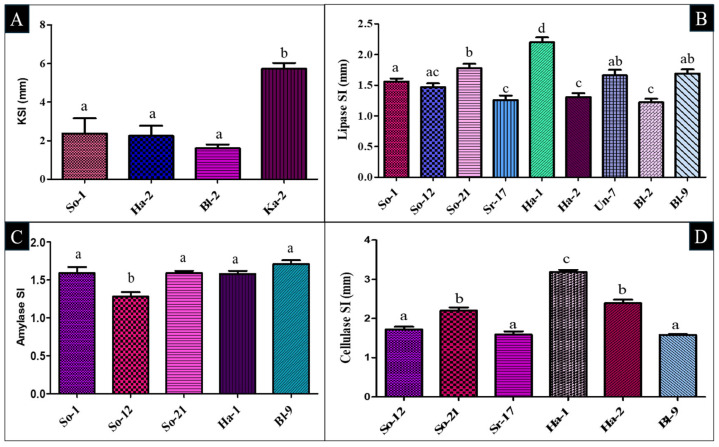
Screening of PGP attributes of all selected isolates was carried out to assess their potential in promoting plant growth through key functional traits: (**A**) KSI, and enzymatic activities such as (**B**) lipase, (**C**) amylase, and (**D**) cellulase zone solubilizing index. Data represents the means ± SD of at least three replicates. Statistical significance was assessed using ANOVA at a 5% significance level (*p* value < 0.05). Different lowercase letters above the bars indicate significant variation, while the same letters show non-significant differences between isolates using the Bonferroni post hoc test at α = 0.05.

Following IAA production, potassium (K) solubilization was identified in 40% of the selected bacterial isolates, with a K-solubilizing index (KSI) ranging from 1.61 to 5.73 mm (Figure S1). Of these, isolate Ka-2 exhibited the highest solubilization activity, forming the largest halo zone, while the lowest activity was found in Bl-2 isolates. Statistical analysis revealed that Ka-2 was significantly different from the other isolates (*p* < 0.05), whereas So-1 showed no significant difference (*p* ≥ 0.05) when compared with Ha-2 and Bl-2 (Figure 2A and Table S3). In addition, 80% of the isolates produced ammonia, suggesting their potential role in biological nitrogen supplementation (Table 1 and Figure S1).

HCN production was found in 50% of the isolates, with maximum intensity observed in Bl-2 and So-1 isolates (Table 1 and Figure S1). In addition, 60% of the bacterial isolates were EPS positive, as evidenced by a mucoid matrix. EPS production has been reported to increase soil aggregation, structure, and water-holding capacity, thus favoring improved plant–microbe interactions under different soil conditions (Table 1 and Figure S1).

Apart from PGP activities, the enzymatic traits of the selected isolates further narrate their potential function in nutrient cycling, organic matter decomposition, and general improvement in soil health. Among the selected isolates, 90% were found positive for lipase activity, with a zone solubilization index (SI) value ranging between 1.22 and 2.20 mm on nutrient agar (NA) medium amended with olive oil. The highest lipase activity was determined in Ha-1 (2.20 ± 0.08 mm), whereas Bl-2 showed the lowest activity, 1.22 ± 0.06 mm (Figure 2B). Amylase production was also seen in 50% of the isolates. The highest amylase SI value was found in So-1 (1.59 ± 0.08 mm) and So-21 (1.59 ± 0.03 mm), and the lowest was for So-12, which is 1.28 ± 0.06 mm (Figure 2C and Figure S1). Cellulase activity was detected in 60% of isolates, with SI values ranging between 1.58 and 3.19 mm on carboxymethyl cellulose (CMC) agar medium (Figure 2D). The highest cellulase activity resulted in Ha-1 (3.19 ± 0.05 mm), and the lowest was shown in Bl-9 isolates (1.58 ± 0.02 mm). Additional information on the screening of hydrolytic enzyme activity among screened bacterial isolates is presented in Table S3 and Figure S1.

### 2.3. Evaluation of Antimicrobial Activity

**Figure 3:**
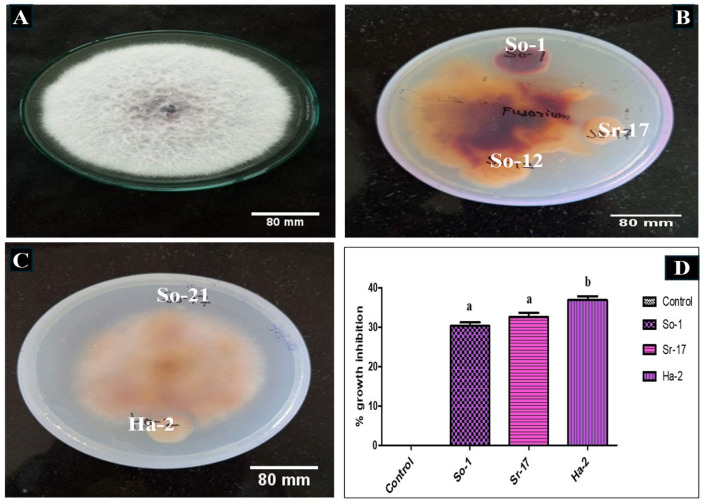
(**A**) Control with normal fungal growth. (**B**,**C**) So-1, Sr-17, and Ha-2 show antagonistic activity against *F. oxysporum* in dual culture assay. Scale bar: 80 mm. (**D**) The percentage inhibition zone of fungal growth by PGPR isolates. Data represents the means ± SD of at least three replicates. Statistical significance was assessed using ANOVA at a 5% significance level (*p* value < 0.05). Different lowercase letters above the bars indicate significant variation, while the same letters show non-significant differences between isolates using the Bonferroni post hoc test at α = 0.05.

In a dual culture plate test, ten selected bacterial isolates were assessed to check their ability to inhibit the growth of *F. oxysporum*. Only three isolates, So-1, Sr-17, and Ha-2, grew on potato dextrose agar (PDA) media and suppressed fungal growth after 5 days of incubation (Figure 3 and Table 1). Following the incubation period, *F. oxysporum* turned orange-red to dark brown colors, indicative of possible metabolic reactions towards inoculating PGPR strains. However, the inhibition zones were comparatively small, where the growth of the fungus was inhibited by 30.4 to 36.9%, and the remaining isolates did not show visible inhibitory activity.

### 2.4. Screening of Bacterial Isolates for pH and Heat Tolerance

**Figure 4:**
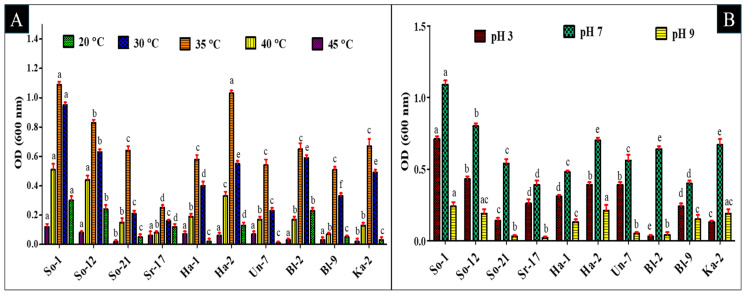
Effect of rhizo-isolates on (**A**) temperature and (**B**) pH. Data represents the means ± SD of at least three replicates. Statistical significance was assessed using one-way ANOVA at a 5% significance level (*p* value < 0.05). Different lowercase letters above the bars indicate significant variation, while the same letters show non-significant differences between isolates using the Bonferroni post hoc test at α = 0.05.

The isolates were assayed to investigate their tolerance against different pH values (3, 7, and 9) and temperature ranges (20–45 °C). So-1 and So-12 grew optimally at all pH levels, indicating their strong adaptability. Four isolates (So-1, So-12, Ha-2, and Un-7) further demonstrated significant growth at pH 3, while five isolates (So-1, So-12, Ha-2, Ka-2, and Bl-9) found moderate growth at pH 9. The findings revealed that the optimal pH for bacterial growth was seven at optical density (OD) 600 nm, with growth reduced at more acidic or alkaline conditions (Figure 4B and Table S4).

On the other hand, nine out of ten isolates had maximum growth at 30 °C and 35 °C, while So-1 and Bl-2 also grew at 20 °C. Isolates’ growth was reduced at 40 °C, indicating reduced bacterial activity at higher temperatures. As a result, physiological analysis revealed that most isolates grew best at 30 °C and 35 °C and pH 7, whereas So-1 and Bl-2 maintained strong growth under varying temperatures (Figure 4A and Table S4).

### 2.5. Molecular Characterization

Due to their temperature, pH tolerance properties, and potential PGP traits, three bacterial (So-1, So-12, and Ha-2) strains were chosen for molecular characterization. A 16S rRNA sequence similarity search showed that all three bacterial isolates belonged to the genera of *Serratia* and *Enterobacter* (Table S5). The isolate So-1 exhibited an identity with the *S. marcescens* strain (accession no. PQ432817.1) of 99.22%. In other cases, the two isolates So-12 (accession no. PQ432822.1) and Ha-2 (accession no. PQ432823.1) showed the highest degree of relatedness with the genus *Enterobacter* and recorded similarity values of 98.66% and 98.99% with *E. cloacae.* A phylogenetic tree constructed based on 16S rRNA gene sequences exhibited that all the bacterial strains were closely affiliated with the *Serratia* and *Enterobacter* bacterial isolates, with *Escherichia coli* as an outgroup. Next to the branches is the %age of replicate trees in which the related taxa are clustered together in the bootstrap test (1000 repetitions).

**Figure 5:**
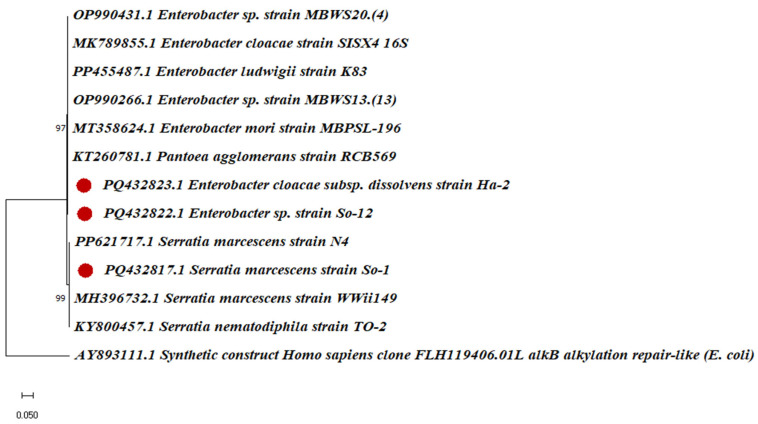
Phylogenetic reconstruction of all three isolated rhizobacterial strains using 16S rRNA gene sequences. Using MEGA software, version 12, a phylogenetic tree was drawn via the neighbor-joining algorithm with a bootstrap value of 1000. The tree is scaled to 0.05 substitutions per nucleotide position. Strains with the red dot were selected for further analysis.

The taxonomic status of the selected isolates was further validated using a phylogenetic tree with their corresponding genera (Figure 5). An isolate like So-1 clustered with *S. marcescens* strain WWii149 (accession no. MH396732.1), whereas So-12 and Ha-2 isolates clustered with *Enterobacter* strain MBWS20.(4) (accession no. OP990266.1) and *E. cloacae* strain SISX4 (accession no. MK789855.1), suggesting probable phylogenetic relationships at the species level. These results confirm accurate identification and reveal the evolutionary relationship of the isolates.

### 2.6. In Vitro Evaluation on Germination and Growth Parameters of Tomato Seedlings

The germination of seeds relies heavily on optimum temperature and water to break exogenous dormancy and induce uniform sprouting. Internal mechanisms like phytohormones also regulate the rate and uniformity of germination. In tomatoes, germination is usually 50% to 85%, resulting in heavy seed loss and economic impact. Enhancing seed germination efficiency reduces waste and improves crop yields through better seedling establishment and plant vigor. In the present in vitro investigation, the seed germination efficacy of three resulting bacterial isolates (So-1, So-12, and Ha-2) was evaluated through eight different treatments. Each isolate was tested at each concentration: 10^6^, 10^7^, and 10^8^ colony-forming units per mL (CFU/mL). The list of treatments and their combinations is given in Table 2.

The findings were positive, with a significant (*p* < 0.05) increase in all parameters measured relative to the control plants. At 10^6^ concentration, the germination of T1 was 80.0 ± 1.00%, which was a significant (*p* < 0.05) rise relative to the control, followed by T2 with a germination rate of 75.6 ± 1.53, a 70.7% rise relative to the control. However, T6 and T7 increased less, with a 4.5% (46.3 ± 1.15) rise by T6 and a 5.2% (46.6 ± 1.00) rise by T7 over control. At 10^7^ concentration, T1 recorded the highest with 92.3 ± 0.58% germination and a notable 108.3% increase over the control, at 44.3 ± 1.53 (Table S6 and Figure S3). T2 recorded 83.3 ± 1.00% germination, which showed an 88.3% improvement, and T3 registered a germination rate of 82.3 ± 0.58%, or an 85.3% rise from the control. T6 also recorded a decline of 7.5% with a 41 ± 1.15% germination rate, while T7 recorded exceptionally, with an increase of 85.3%, recording a germination rate of 82.3 ± 1.53%.

**Figure 6:**
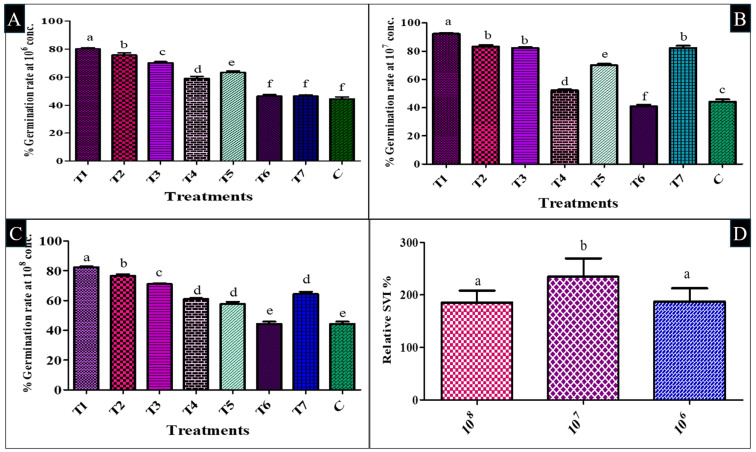
Impact of PGPR strains on in vitro tomato seed germination at three different concentrations: (**A**) 10^6^; (**B**) 10^7^; (**C**) 10^8^; and (**D**) SVI at various concentrations of bacterial suspension. Data represents the means ± SD of at least 3 replicates. Statistical significance was assessed using one-way ANOVA 5% highly significant (*p* value < 0.05). Different lowercase letters above the bars indicate significant variation, while the same letters show non-significant differences between isolates by using the Bonferroni post hoc test at α = 0.05.

At the concentration 10^8^, T1 again recorded the highest germination rate of 82.3 ± 0.58%, which registered an increase of 85.3% over the control. T2 resulted in 76.6 ± 1.00% germination, indicating a 72.3% increase, and in T3 treatment, the germination was observed at 71 ± 0.58%, resulting in a 60.3% increase. In contrast, T6 had no increase in germination %age, at 44.3 ± 1.53%, while T7 performed better with a 45.3% increase, and a germination rate of 64.3 ± 1.53%. Overall, T1 consistently had the best of all concentrations and had the most significant increase in germination over the control. T3 and T2 had notable improvement at varied concentrations, and T6 exhibited declining trends at 10^7^ but not 10^8^ concentration (Figure 6A–C and Table S6).

In parallel with seed germination, seedling vigor index (SVI) further revealed significant results over the control across all dilution levels (10^6^, 10^7^, and 10^8^). Control treatment showed the lowest SVI values, which were 193.4, respectively, and thus was set as the baseline (100%) for comparison. In the 10^6^ concentration, T1 treatment recorded the highest SVI (610.4), with a Relative SVI of 315.6%, followed by T2 (SVI: 486.4; Relative SVI: 251.4%) and T3 (SVI: 436.3; Relative SVI: 225.6%). Other treatments, such as T4 (289.1; 149.5%), T5 (343.9; 177.8%), T6 (262.4; 135.7%), and T7 (274.9; 142.1%), also showed higher vigor, statistically significant (*p* < 0.05) compared to the control. In the 10^7^ dilution, T1 again had the highest SVI (732.2) and Relative SVI (378.6%), followed by T2 (599.8; 310.1%), T3 (565.1; 292.3%), and T7 (545.9; 282.2%). T5 (457.3; 236.5%), T4 (291.1; 150.5%), and T6 (240.5; 124.4%) also surpassed the control.

At the 10^8^ concentration, T2 showed the highest SVI (520.9) and Relative SVI (269.3%), closely followed by T1 (515.7; 266.5%) and T3 treatment (430.7; 222.6%). Other treatments, such as T7 (381.5; 197.3%), also exceeded the control. These results indicated that all treatments within their respective concentration significantly (*p* < 0.05) improved SVI over the control, with T1 and T2 consistently producing the highest SVI and Relative SVI values across all dilutions, demonstrating their strong positive effects on seedling growth. In contrast, when comparing concentration treatments with other treatments, only the 10^7^ CFU/mL dilution shows significant results (Figure 6D and Table S6).

**Figure 7:**
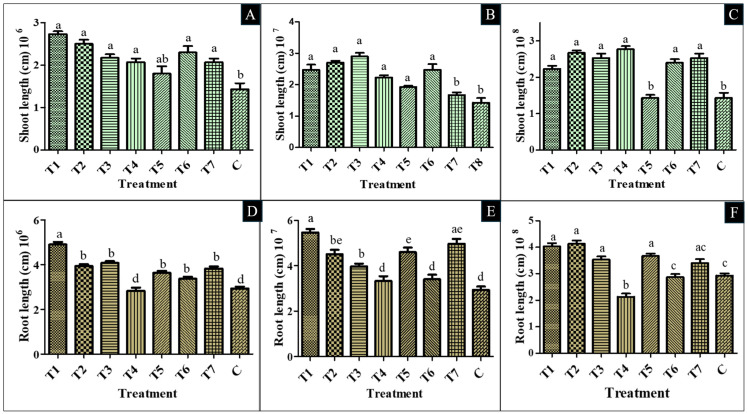
Effect of rhizobacterial strains at different concentrations on (**A**–**C**) tomato root length; and (**D**–**F**) shoot length of tomato seedling. Data represents the means ± SD of at least 3 replicates. Statistical significance was assessed using ANOVA 5% highly significant (*p* value < 0.05). Different lowercase letters above the bars indicate significant variation, while the same letters show non-significant differences between isolates by using the Bonferroni post hoc test at α = 0.05.

Following germination, the root length measurement indicated that T1 exhibits the highest growth performance among all the tested concentrations. At the 10^6^ concentration, T1 shows a significant root length of 4.90 ± 0.20 cm, a 67.2% rise over the control. At 10^7^, the root length was 5.47 ± 0.15 cm, an 86.6% increase relative to the control, and at 10^8^, the root length was 4.03 ± 0.21 cm, representing a 37.4% increase compared to the control (T8). At the 10^6^ concentration, T2 further revealed significant results, with an increase of 34.1% and a root length of 3.93 ± 0.15 cm. At the 10^7^ concentration, the length of the roots was 4.50 ± 0.20 cm, with a rise of 53.8% over the control. At the 10^8^ concentration, the root length was detected as 4.13 ± 0.21 cm with a rise of 41.0%, respectively. In addition, T3 displayed moderate improvement, particularly at 10^6^ and 10^7^, with increases of 38.8% and 42.1%. T4 treatment further showed the lowest root length, with reductions of 3.4% (2.83 ± 0.25 cm) at 10^6^ and 27.3% (2.13 ± 0.21) at 10^8^ (Figure 7A–C). Overall, T1 and T2 treatments resulted in maximum root growth, while T4 consistently performed lower results (Table S6).

After the root length analysis, the shoot length result further showed that T1 performed significantly at all concentrations. At 10^6^, the shoot length of T1 was 2.73 ± 0.12 cm, an increase of 90.2% compared to the control (1.43 ± 0.25 cm). At 10^7^, the shoot length of T1 was 2.47 ± 0.29 cm, an increase of 72.7%, and at the 10^8^ concentration, the seedling shoot length was 2.23 ± 0.15 cm, an increase of 55.1%. Similarly, T2 and T3 also resulted in improvements, especially at 10^6^ and 10^7^ concentrations, respectively. At the 10^6^ concentration, the T2 shoot length exhibited a 74.8% increase, with a shoot length of 2.50 ± 0.17 cm over the control. On the other hand, at the 10^7^ concentration, the T3 shoot length detected was 2.90 ± 0.20 cm, showing a 68.8% enhancement over the control (Figure 7D–F and Table S6). To follow up with these statements, T1 and T2 notably resulted in the maximum root and shoot growth across all the concentrations.

### 2.7. In Vivo Polyhouse Screening and Measuring the Growth Parameters of Tomato Seedlings

Based on the in vitro seed germination test, which was conducted at three different concentrations (10^6^, 10^7^, and 10^8^ CFU/mL), the findings revealed that the 10^7^ CFU/mL bacterial suspension was one of the most effective, and it was further used for the subsequent in vivo experiment. The in vivo pot trial experiment was conducted for 45 days on a tomato plant, in which parameters like % germination, root-shoot length, and fresh and dry biomass were measured.

**Figure 8:**
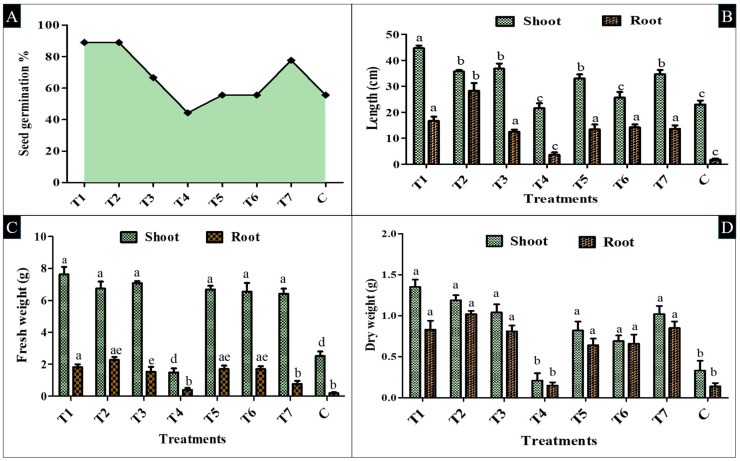
Effect of PGPR bacterial strains on (**A**) seed germination; (**B**) shoot and root length; (**C**) fresh shoot and root; and (**D**) dry weights of tomato seedlings after a 45-day trial period under in vivo polyhouse conditions. Data represents the means ± SD of at least 3 replicates. Statistical significance was assessed using ANOVA 5% highly significant (*p* value < 0.05). Different lowercase letters above the bars indicate significant variation, while the same letters show non-significant differences between isolates by using the Bonferroni post hoc test at α = 0.05.

**Figure 9:**
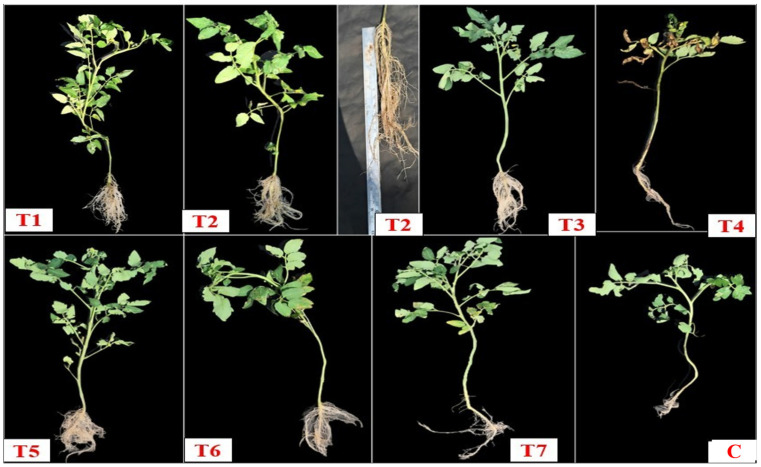
Effect of PGPR bacterial strains on shoot and root length of tomato seedlings at 45 days after sowing under polyhouse conditions. Lettering in the figures refers to different bacterial strains.

Regarding germination performance, the %age varies between 44.3% and 89%. Interestingly, T1 and T2 treatments exhibited the highest values of germination at 89%, with a rise of 60% over the control, whose germination ability was only 55.6%. Similarly, T7 further showed seed germination efficiency, which was 77.6 ± 0.58%, a 39.57% increase compared to the control (Figure 8A). Meanwhile, T2 increased the root length (28.3 ± 2.98 cm) by over 1560%, and the shoot length (35.7 ± 0.57 cm) increased by 55% against the control. T1 increased the shoot (44.7 ± 0.96 cm) by 94%, and root length (16.7 ± 1.56 cm) increased by 882% over the control (1.7 ± 0.47 cm) (Figure 8B and Figure 9).

In line with the improvements observed in seedling length, the fresh and dry biomass of the plants further showed a similar enhancement pattern. In terms of fresh weight, a significant shoot fresh weight was found in T1, which was 7.63 ± 0.47 g, and the root fresh weight was 1.81± 0.18 g, 805% higher than the control root fresh weight (0.20 ± 0.05 g). Similarly, T2 showed a 6.74 ± 0.44 g shoot fresh weight, and the plant root fresh weight was 2.27 ± 0.18 g, which are 1000% increases over the control root length weight, respectively (Figure 8C).

In comparison with fresh weight, the plant dry biomass was further revealed to be significantly (*p* < 0.05) enhanced over the control. In T2 treatment, the shoot dry biomass was 1.19 ± 0.06 g, a 260% increase, and the root biomass was 1.02 ± 0.04 g, which is 629% higher than the control, whose root dry weight was 0.14 ± 0.04 g, and the shoot dry weight was 0.33 ± 0.12 g. Similarly, T1 also showed significant biomass accumulation, in which the shoot dry mass was 1.35 ± 0.09 g, an increase of 309%, and the root dry weight was 0.83 ± 0.11 g, 492% higher than the control seedlings (Figure 8D and Figure 9). Overall, these in vivo results are aligned with the present in vitro findings, where *S. marcescens* So-1 (T1) and *Enterobacter* sp. So-12 (T2) strains exhibited notable performance across all parameters.

In contrast, where all treatments revealed notable improvement, the consortium treatment T4 (T1+ T2 + T3) recorded the lowest seed germination %age, which was 44.3% over the control. This consortium also caused appreciable decreases in shoot length, which was 21.6 ± 1.82 cm, root length 3.6 ± 0.93 cm, and shoot fresh weight, which was 1.48 ± 0.26 g, showing a 41% reduction compared to the controls and indicating potential antagonistic interactions between the isolates. Overall, the present results revealed that inoculation with single bacterial isolates, particularly *S. marcescens* So-1 (T1) and *Enterobacter* sp. So-12 (T2), significantly (*p* < 0.05) promotes early seedling growth and plant biomass yield under polyhouse conditions at the 10^7^ CFU/mL concentration formulation. These indigenous strains have the potential to enhance early plant growth stages and possess good potential for use in sustainable tomato production.

Following biomass parameters, the ANOVA test showed significant increases (*p* < 0.05) in all the agronomic parameters compared to the control. Pearson correlation analysis was performed among parameters like shoot-root length, fresh weight, and dry weight (Table 3). These results demonstrate strong positive correlations, particularly between shoot length and shoot dry weight (r = 0.920) and root dry weight (r = 0.874), supporting the interdependence of early growth parameters and their potential predictive value for productivity.

### 2.8. Chlorophyll Estimation

**Figure 10:**
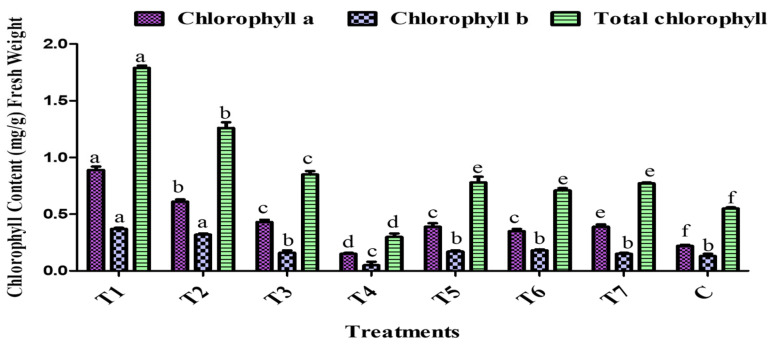
The effect of inoculation with PGPR strains on the chlorophyll content in tomato leaves after 45 days of treatment under polyhouse conditions. Data represents the means ± SD of at least 3 replicates. Statistical significance was assessed using ANOVA 5% highly significant (*p* value < 0.05). Different lowercase letters above the bars indicate significant variation, while the same letters show non-significant differences between isolates by using the Bonferroni post hoc test at α = 0.05.

The chlorophyll content of tomato leaves was significantly affected by the different PGPR treatments. Chlorophyll content was quantified after 45 days of plant growth under polyhouse conditions. As shown in Figure 10, T1 has the highest total chlorophyll content (1.79 ± 0.02 mg/g), followed by T2 (1.26 ± 0.05 mg/g), which is a 129.09% increase over the control, indicating enhanced photosynthetic potential. Meanwhile, the T4 treatment recorded the lowest chlorophyll levels (0.30 ± 0.03 mg/g), showing a 45.4% reduction over the control (Figure 10). In parallel, the control shows moderate chlorophyll content (0.55 ± 0.01 mg/g). Treatments T5 and T7 further resulted in improved pigment levels over the control. Overall, the T1 and T2 PGPR bioformulation significantly promoted chlorophyll biosynthesis, while the T4 consortium negatively affected leaf pigmentation, which is aligned with the in vivo and in vitro results.

## 3. Discussion

With the increasing adverse effects of chemical fertilizers on soil health, farmers are now shifting towards sustainable farming practices. Due to this context, PGPR have been identified as eco-friendly biofertilizers that can increase crop yield as well as soil fertility under various environmental stresses [35]. The present study explored the PGP potential of native PGPR bacterial isolates obtained from various soils in the Lower Western Himalayan Zone of Himachal Pradesh, India. A total of 112 distinct rhizobacterial isolates were obtained from these locations using the serial dilution technique. Out of these, only 10 bacterial isolates were selected and further screened for growth-promotion activities, as well as their ability to tolerate various environmental stresses and promote plant growth.

### 3.1. Evaluation of In Vitro Environmental Stress Tolerance of Bacterial Isolates

PGPR can promote plant growth through several mechanisms, including the production of phytohormones, solubilizing nutrients, and increasing the plant defenses against pathogens. Out of these, IAA is an important phytohormone with the capacity to promote cell differentiation, seedling growth, and the elongation and formation of lateral roots in plants [36]. The present finding revealed that all the tested bacterial isolates produced IAA in the range of 2.6 to 24.1 µg/mL using the Salkowski colorimetric assay. These results are aligned with Kaur and Sharma [37], who showed that PGPR strains produced IAA (53.1–71.1 μg/mL) under favorable growth performance, whereas Kalimuthu et al. [38] noted that tomato rhizobacterium produced maximum levels of bacterial IAA production (12.80 µg/mL when 0.7% L-tryptophan). Also, Lata et al. [39] reported in their study that the highest IAA was produced on isolated GAC-2 with 34.56 μg/mL, followed by isolate GAC-91 (33.88 μg/mL) and isolate GAC-118 (33.37 μg/mL).

Similarly, the production of ammonia was detected in 50% of the rhizo-isolates. This trait is the principal source of nitrogen (N) and therefore facilitates root and shoot growth, as well as subsequent biomass production [40]. The findings align with Khan et al. [41], whose findings stated that fifteen isolates produced ammonium positively. In this regard, Alonazi et al. [42] explained that 82% of the bacterial isolates were potential ammonium producers. In addition to ammonium production, potassium solubilization activity was further observed in 40% of the isolates. Among them, the isolated Ka-2 has the highest KSI with 5.73 ± 0.30 mm, followed by So-1, whose KSI is 2.38 ± 0.78 mm. Potassium is needed in plants for metabolism, including the synthesis of cells and the formation of enzymes, protein, and cellulose. Potassium also plays a role in regulating the transport of water and nutrients through the xylem [43]. This is in alignment with Azizah et al. [43], who reported that KF668 isolates show a clear zone around the colony. Whereas Zhao et al. [44] revealed in their study that forty-nine of the screened isolates demonstrated the ability to increase potassium solubility, with solubilization indices ranging from 1.16 to 3.71.

In addition, 60% of the bacterial isolates were EPS positive, as marked by the evidence of a mucoid matrix. Meanwhile, HCN production was found in 50% of the isolates. Among these isolates, Bl-2 followed by So-1 isolates show the maximum intensity of HCN synthesis. HCN is also an important PGP trait that protects plants from pathogens such as fungi or others. These results are aligned with Abd El-Rahman et al. [45], who reported that out of 39 isolates, only 6 isolates were able to produce HCN.

Apart from PGP activity, the enzymatic activity of the PGPR further shows their potential in nutrient cycling, organic matter decomposition, and general improvement in soil health by breaking down complex organic substrates. To align with this, the present study shows that 90% of the isolates possessed lipase activity, 50% amylase, and 60% had positive cellulase activity, with SI ranging from 1.58 to 3.19 mm. A similar study was performed by Mekonnen et al. [46], which showed that out of three isolates, only two were positive for amylase activity, while all were positive for cellulase and lipase activity.

The present findings are aligned with Zhao et al. [44], who reported that the optimal pH for bacterial growth is 7 and 9. Fasusi et al. [24] found that the optimum temp. is about 30 °C and 35 °C.

### 3.2. Antagonistic Activity of the Isolates

In the present work, antagonistic activity was examined, particularly against *F. oxysporum*, due to its economic importance as the pathogen causing vascular wilt of tomato, a major disease leading to heavy loss in tomato yield worldwide [19]. Our work confirmed that among the ten isolates, three isolates showed an antagonistic effect against *F. oxysporum*, with inhibition of 30.4 to 36.9%. This indicates that rhizobacterial strains used in the present work have moderate to acceptable biocontrol activity against the soilborne fungal disease of the tomato plant. This is in agreement with the Yao et al. [47] work, wherein 11 PGPR strains exhibited different levels of antagonistic activity against *F. oxysporum*, with inhibition varying from 20.80 ± 1.47% to 73.57 ± 1.15%.

While the strains were not tested against other phytopathogens in this phase of the work, several earlier studies have demonstrated that genera such as *Serratia* and *Enterobacter* often possess broad-spectrum antifungal properties, including activity against pathogens like *F. graminearum* and *F. moniliforme* [48], as well as *Rhizoctonia solani* [49]. In addition, Chakraborty et al. [50] reported that *S*. *marcescens* show their antagonistic effects against pathogens such as *Poria hypobrunnea*, *Fomes lamaoensis*, *Sclerotium rolfsii*, and *Ustulina zonata*, with inhibition ranging from 39 to 74% on solid media and 24 to 59% in liquid cultures. These findings underscore the potential of *S. marcescens* and related strains for broad biocontrol applications.

### 3.3. Bacterial Identification and Its Pathogenicity

Due to their temperature and pH tolerance properties, along with potential PGP traits, three bacterial (So-1, So-12, and Ha-2) strains were chosen for molecular characterization. A 16S rRNA sequence similarity search showed that all three bacterial isolates belonged to the genera of *Serratia* and *Enterobacter*.

While some of these strains of genera, particularly those in clinical settings, have been reported to be opportunistic pathogens, caution should be taken in distinguishing non-pathogenic from pathogenic strains. The *S. marcescens* So-1 and *Enterobacter* sp. So-12 or Ha-2 strains employed in this work were isolated from the healthy tomato rhizosphere and were selected particularly for their plant growth-promoting properties. Preliminary biosafety assessments did not reveal any hemolysis, which confirmed their non-pathogenic status. In line with Zaheer et al. [51] and Abreo and Altier [52], our *S. marcescens* So-1 strain produced orange to maroon pigmentation, a trait that is consistent with the environmental production of prodigiosin and known to genetically differentiate these strains from non-pigmented clinical isolates that are stereotypically implicated in causing human infections. Sharma et al. [53], Zhang et al. [54], Almaghrabi et al. [55], Patel et al. [49], and Singh and Jha [48] have reported the safe use of *Serratia* and *Enterobacter* strains in promoting plant growth in abiotic stress, further confirming their potential as efficient bioinoculants. With the increased number of *Serratia* and *Enterobacter* strains that have been successfully employed in agriculture, we believe that with rigorous strain-level screening and adherence to regulatory requirements, the isolates reported in this study hold realistic and environmentally friendly promise for sustainable crop production.

### 3.4. In Vitro- and In Vivo-Level Investigation of PGPR Inoculant to Enhance Tomato Plant Growth

After identification, Durga Abhinav tomato seeds were inoculated with bacterial cultures at three concentrations (10^6^, 10^7^, and 10^8^ CFU/mL) and further evaluated in vitro for their efficacy on germination and seedling length. The germination rate, SVI, and root-shoot length were found to be enhanced considerably (*p* < 0.05) in T1 compared to the control. T1 and T2 induced germination of seeds from 83.3% to 108.3% and increased SVI by up to 732.2% compared to the control at a concentration of 10^7^. The present study observation was in concordance with the Zhao et al. [56] study, where T8 showed the highest effect on the tomato germination percentage, which was increased by 99.9% compared to the control. R25 and R41, with low seedling promotion effect, were excluded, and the two highest seedling index combinations (R62 + R219 + R317 + R325 (T1) and R62 + R219 + R317 (T2)) were achieved. Fiodor et al. [57] further observed that *S. marcescens* AF8I1 showed significant enhancement in the germination of carrot seeds by 156.88%. Similarly, González-Ista et al. [58] further observed that among all concentrations, 10^5^ CFU/mL of the *Enterobacter* sp. L7 showed a significant increase in fresh weight of roots (100%) and shoots (69%) compared to *Serratia* sp. H6 (61% and 59%).

The in vivo PGPR inoculation results further revealed that T1, followed by T2, showed remarkable performance among all the tested parameters. For germination, the T1 and T2 treatments showed the highest value of germination at 89%, with a 60% increase over the control. Meanwhile, T2 increased the root length (28.3 ± 2.98 cm), and the shoot length (35.7 ± 0.57 cm) increased by 55% against the control. A similar study conducted by Mekonnen et al. [46] reported that BDUA1 and BDUA2 isolate treatments caused a notable difference in plant height of both the tomato varieties (Melkesalsa variety and Maya variety) when compared to the control. In the Maya variety, maximum plant height was observed with BDUA1 (42.25 cm) inoculation, followed by BDUA2 (38.65 cm) inoculation. Furthermore, Melkesalsa inoculation with BDUA1 showed the tallest height (36.91 cm), followed by BDUA2 (31.74 cm) inoculation. Consortium treatments for BDUA1 and BDUA3 showed minimal effect for both the tomato varieties. The findings of the present study are consistent with Mengistie and Awlachew [59], who reported in their results that tomato variety Kochero seedlings treated with B1 indicated the highest shoot length (34.86 ± 3.1 cm), shoot fresh weight (10.86 ± 0.4 g), shoot dry weight (8.33 ± 0.76 g), root length (12.0 ± 0.0 cm), root fresh weight (3.0 ± 0.06), and root dry weight (1.2 ± 0.08). Kalimuthu et al. [38] found that the shoot length was 12 cm, fresh and dry weights were 1.80 g and 0.14 g, and there was 80% seed germination when tomato plants were inoculated with *Pseudomonas* after a 30-day trial period.

On the other hand, where T1 and T2 have significant effects, the T4 consortium had relatively lower efficacy than the control, perhaps through an antagonistic interaction among the strains. Although our in vitro compatibility test for our strains of *S. marcescens* (So-1), *Enterobacter* spp. (So-12), and *Enterobacter* spp. (Ha-2) did not show antagonism among the strains, the co-inoculated treatment did not result in enhanced plant growth or nutrient uptake. It suggests that compatibility in vitro is not always indicative of functional synergy in the soil. Microbial strains competing for limiting colonization sites, nutrient sources derived from root exudates, or ecological niches in the rhizosphere may be the cause. Such subtle interactions can hinder the establishment and cooperative activity of inoculated strains. Interference in microbial signaling pathways like quorum sensing or hormonal crosstalk may also suppress the expression of key growth-promoting phenotypes like IAA production, phosphate solubilization, or ACC deaminase activity.

Moreover, the host plant response and the effect of the organic biostimulant may have been responsible for the lack of additive effects. The modification of root exudation by nutrient levels or feedback technology may limit microbial recruitment or reduce compatibility. This argument is supported by Singh et al. [60] and Rosier et al. [61], who established that the PGPR consortium can induce competition rather than cooperation in plant systems. Abou Jaoudé et al. [62] also established that microbial interactions in the rhizosphere were highly context-dependent and controlled by plant-mediated selection pressure. Similar findings were also presented by Mekonnen et al. [63], He et al. [64], and Myresiotis et al. [65]. Mekonnen et al. [63] demonstrated that single inoculants of *Pseudomonas* and *Bacillus* enhanced the growth of tomatoes, but some of the consortium treatments under greenhouse conditions were not as good as single inoculants because of probable ecological interference. Their observation is consistent with the fact that not all strain combinations enhance performance, particularly under controlled conditions. He et al. [64] and Myresiotis et al. [65] also described reduced efficacy in PGPR mixtures because of inhibitory compounds or metabolic interaction incompatibility. The observations are consistent with our hypothesis that the reduced efficacy of T4 was most probably because of subtle competitive or physiological interactions among microbes in the rhizosphere.

Generally, the present findings indicate excellent positive correlations, i.e., between shoot length and shoot dry weight (r = 0.920) and root dry weight (r = 0.874), corroborating the interdependence of early growth parameters and their possible predictive potential for enhanced productivity. Similarly, the chlorophyll content of tomato leaves was significantly affected by the different PGPR treatments. T1 had the highest total chlorophyll content (1.79 ± 0.02 mg/g), followed by T2 (1.26 ± 0.05 mg/g). In alignment with this, Patani et al. [66] reported a significant rise in the chlorophyll of the leaves of tomato plants in NCT4, NCT1, LCT4, LAT3, and LBM4 compared to those of untreated plants.

Among all the parameters measured, the present results confirm that single PGPR strains (T1 and T2), particularly when applied at the best dose-dependent concentrations, notably enhance tomato seedling growth. This paper, therefore, presents new insights into precision bioinoculant applications and indirectly promotes sustainable farming.

### 3.5. Future Recommendations

The present study followed a routine protocol by conducting a 45-day polyhouse trial to evaluate the early-stage vegetative response to the PGPR inoculation of tomato plants, as performed by Almaghrabi et al. [55] and Mengistie and Awlachew [59]. Although the improvements were impressive in shoot-root growth, biomass accumulation, and chlorophyll content, future studies must go beyond vegetative to flowering and fruiting stages under polyhouse as well as open-field conditions. Such trials are crucial to directly evaluate yield-related traits and determine the agronomic value of PGPR treatments. Given the known variability of the PGPR–host genotype interaction, confirmation in a larger set of tomato cultivars, like determinate, indeterminate, hybrid, and open-pollinated varieties, as well as other solanaceous crops like capsicum and brinjal, is strongly advised. For biosafety and regulatory acceptability determination, whole-genome sequencing of the most promising strains, such as *S. marcescens* So-1 and *Enterobacter* spp. Ha-2 and So-12, must be conducted to identify key plant-beneficial genes and eliminate any virulence or resistance determinants. Further, while Salkowski’s reagent was a convenient initial aid in auxin detection, as suggested by Lata et al. [39] as well as Dhole et al. [67], future studies must employ more specific analytical techniques like HPTLC or HPLC for the quantitative estimation and profiling of indole compounds. Finally, with the limitation of culture-based approaches, molecular tools like 16S rRNA sequencing and metagenomics must be employed to unlock the full diversity and functional potential of phosphate-solubilizing microbes, as reported by Hegyi et al. [68], Brar et al. [69], and Qingwei et al. [70]. Stewart [71] found that not all soil bacteria are culturable in lab conditions.

## 4. Materials and Methods

### 4.1. Soil Sampling and Rhizobacterial Isolation

#### 4.1.1. Description of Study Area

Rhizosphere soil samples were taken from healthy tomato plants cultivated in different districts of the Lower Western Himalayan Zone of Himachal Pradesh, namely Kangra, Hamirpur, Una, Bilaspur, and the lower regions of Solan and Sirmaur (Table S1). These areas are mostly characterized by low hill soil, which are usually grayish brown in color and texture from silty loam to loamy and sandy loam at elevations of between 350 and 1000 m above mean sea level. Such well-drained soils, combined with a pH level of 6.2 to 7.3 [72], are suitable for both off-season and seasonal tomato farming [13]. They have a total nitrogen range of 281.84 to 260.48 kg ha^−1^, a total phosphorus range of 18.70 to 10.54 kg ha^−1^, and a total potassium range of 152.47 to 122.27 kg ha^−1^. The electric conductivity of soil ranged from 0.36 to 0.10 dS m^−1^. The water-retaining capacity of soil samples ranged from 46.8% to 37.7%.

#### 4.1.2. Collection and Isolation of Rhizobacteria

For sampling, rhizosphere soil samples were taken from healthy tomato plants. The plants were carefully uprooted from each of the selected locations. Loosely attached bulk soil was shaken off gently to only keep the soil closely adhered to the roots. This rhizospheric soil was collected from a depth of more than 5 cm. Each sample was placed in a sterile, tagged plastic bag, taken to the Microbiology Laboratory, and stored at 4 °C for further processing.

To isolate rhizobacteria, 1 g of each rhizospheric soil sample was diluted serially to 10^−8^. Suitable dilutions were pour-plated onto PVK agar medium and incubated at 28 ± 2 °C for 48–72 h [73]. Morphologically distinct bacterial colonies showing halozones were selected and further purified by re-streaking onto different NA plates. The pure bacterial isolates were stored on nutrient agar slants at 4 °C for further biochemical characterization and analysis.

### 4.2. Biochemical Characterization

Biochemical tests were conducted to identify the bacterial isolates’ various enzymatic and metabolic capabilities. The number of biochemical tests is mentioned below.

#### 4.2.1. Catalase and Oxidase Test

A drop of 3% hydrogen peroxide (H_2_O_2_) was added to a glass slide inoculated with bacterial inoculum, as per Khan et al. [41]; any instant bubble formation was considered a positive catalase reaction. In the oxidase test, 24 h old cultures of NA were employed; 2–3 drops of α-naphthol reagent were directly added to colonies, and a purple-blue color in 5–10 s was positive, as per Singh et al. [36].

#### 4.2.2. Mannitol Fermentation Test

In the mannitol fermentation test, mannitol salt agar was inoculated with bacterial cultures and incubated at 37 °C for 24–48 h. Growth and color change from pink to yellow showed salt tolerance and confirmed mannitol fermentation [74].

#### 4.2.3. Citrate Utilization Test

The citrate test was performed by streaking isolates on Simmons’ citrate agar slants and incubating at 37 °C for 24 h; a green to blue color change was positive for citrate utilization as the only carbon source [75].

#### 4.2.4. Urease Test

For the urease test, the isolates were grown on urea agar and incubated at 28 ± 2 °C for 24 h; a pink or red color showed urease activity due to ammonia production, as per the method of Hiranmayee et al. [76].

#### 4.2.5. TSI Test

The TSI test was performed by stabbing the butt and streaking a TSI agar slant with the test organism, then incubating at 37 °C for 24 h. A yellow slant and butt indicated glucose, lactose, and/or sucrose fermentation, and a red slant with yellow butt indicated glucose-only fermentation. Gas production was observed as cracks or bubbles in the agar, while black precipitate formation indicated hydrogen sulfide (H_2_S) production [76].

#### 4.2.6. MR Test

For the MR test, 20 µL of 18–24 h old freshly prepared bacterial culture was inoculated into nutrient broth (NB) and incubated at 37 °C for 24 h. Then, 1 mL of culture was poured into a sterile test tube, and 2–3 drops of methyl red indicator were added. A red color was positive, which showed stable acid production due to glucose fermentation [77].

### 4.3. In Vitro Bioassays to Evaluate Growth-Promoting and Biocontrol Attributes of the Rhizobacterial Isolates

#### 4.3.1. Characterization of PGPRs Based on Their PGP Capabilities

The PGP properties of rhizobacterial isolates were assessed through the following characterizations:

##### Qualitative Screening of K Solubilization Activity

Each rhizo-isolate was qualitatively analyzed for its K-solubilization ability on Aleksandrow’s agar medium [78]. The isolates were inoculated at the center of the agar medium and maintained at 28 ± 2 °C for 15 days. The formation of a halozone surrounding the colony indicated positive potassium solubilization activity [79].

##### Qualitative Estimation of Ammonia Synthesis

The bacterial culture was inoculated in peptone water (1 g/L peptone and 4.3 g/L sodium chloride), and the tubes were incubated at 28 ± 2 °C for 48 to 72 h. After incubation, 0.5 mL of Nessler’s reagent was added to each tube. Yellow to brown coloration indicates the production of ammonia [80].

##### Screening of EPS Formation

Luria Bertani (LB) agar was prepared for EPS screening, and the pH was set to 7 using a digital pH meter before sterilization. Following sterilization, sterile Whatman filter paper, 6 mm in diameter, was transferred onto solidified LB agar containing 10% sucrose. A 2 µL aliquot of a 24–48 h old, selected isolate culture was plated on the filter paper, and the plates were maintained for 2 days at 30 °C. Mucoid colonies near the filter paper after incubation were observed, reflecting exopolysaccharide production [81].

#### 4.3.2. Characterization of PGPRs Based on Their Biocontrol Activity

Each selected rhizo-isolate was examined for the development of HCN activity by employing the standard procedure [82]. This method introduced fresh bacterial isolate cultures into NB medium enriched with glycine (1.4 g/L). No. 1 Whatman filter paper pieces/strips were cut and put in the test tubes to avoid contact with the medium. The pieces were immersed in picric acid with 0.5% and 2% sodium carbonate (Na_2_CO_3_) and kept at the top of the test tubes. The test tubes were covered with parafilm and maintained at 28 °C for 5 to 7 days. After incubation, the formation of a deep yellowish to orange or orange to brown coloration revealed HCN production by the rhizosphere isolates.

#### 4.3.3. Qualitative Characterization of PGPRs Based on Their Hydrolytic Enzyme Production Activity

The hydrolytic enzyme production activities of bacterial isolates can be assessed through the following characterizations:

##### Lipase Activity

The lipase hydrolytic-enzyme production activity was determined according to the standard procedure [83]. This method introduced 4–5 drops of olive oil to the NA medium, and selected isolates were grown on the prepared agar medium. The agar plates were maintained at 28 ± 2 °C for 2–3 days. The plates treated with isolates were then filled with copper sulfate (CuSO_4_) solution and left untouched for 10–15 min. The greenish-blue color zone appearance around the colonies of bacteria indicated lipase production. Hydrolytic enzyme activities were quantified by applying the following formula given by Yasmin et al. [84]:

SI = Diameter of the bacterial colony (mm) + Colony halozone diameter (mm)/Diameter of the bacterial colony (mm)

##### Amylase Activity

The bacterial culture was spotted at the center of the starch agar medium plates and maintained for 48 h at 30 °C [83]. After incubation, the plates were flooded with Lugol iodine solution, left for 4–5 min, and then the iodine solution was discarded. The colorless zones surrounding the colonies exhibited the formation of amylase activity.

##### Cellulase Activity

Cellulase enzymes were analyzed using an M9 agar medium amended with CMC as a carbon source, and 1.2 g of yeast extract/L was added [83]. Plates were inoculated with fresh bacterial culture and maintained at 28 ± 2 °C for 5 days [85]. Isolates with clear halozones around the colonies are a positive sign for cellulase formation.

#### 4.3.4. Characterization of PGPRs Based on Their Phytohormone Production Activity

IAA formation was assessed according to the method given by Ganesh et al. [86], with slight modifications. A 50 mL LB broth medium was supplemented with 0.1% DL-tryptophan as the IAA precursor. The sterilized medium was introduced with 500 µL of fresh culture and maintained at 250 rpm in a shaker incubator for 5 to 7 days at 30 °C. Fully grown cultures were centrifuged at 10,000 rpm for 10–15 min, then 2 mL of the bacterial supernatant was dispensed into 4 mL of the Salkowski reagent. Then, 2–3 drops of orthophosphoric acid were applied, and the mixture was placed in the dark to allow color development. After incubation, pink coloration in the test tubes was a positive indication for IAA development. Optical density (OD) at 530 nm was measured using a UV-Vis spectrophotometer after 1 to 2 h. IAA concentrations were checked using the IAA standard curve with a range of 0 to 100 μg/mL in LB medium, and the data were presented as µg/mL relative to the control.

### 4.4. Evaluation of Antimicrobial Activity

The antimicrobial potential of the selected PGPR isolates was observed against fungal isolates, viz., *F. oxysporum* (SR266–9), using a dual culture procedure reported by Agarwal et al. [87]. This method transferred a 5 mm agar plug from the edge of 5- to 7-day-old fungal cultures to the center of PDA agar medium plates. A fresh loopful of bacterial culture was inoculated 2 cm away from the fungal pathogens. The control Petri plates were also stabilized by putting the fungal pathogen on the PDA agar medium plate without the tested bacteria. The treated and non-treated plates were maintained for 5 to 7 days at 28 °C [24]. Each treatment was conducted with three individual replicates. After the incubation period, the giving zone of the bacterial isolates against the fungal culture was regarded as effective against the fungal isolate. The radial growth inhibition percentage of *F. oxysporum* was determined by using the following formula:

% growth inhibition: Fungus growth in control-fungus growth in treated sample/ Fungus growth in control × 100

### 4.5. Bacterial Tolerance Response to Different pH and Heat

Bacterial isolates’ tolerance towards different pH levels was evaluated by preparing LB broth. The pH of the medium was adjusted to alkaline, neutral, and acidic (3, 7, 9) by utilizing 1 M hydrochloric acid (HCl) or 1 M sodium hydroxide (NaOH). After the pH of the medium was fixed, the broth was autoclaved at 121 °C for 15 min. Aseptically, exactly 30 µL of each overnight bacterial culture was transferred to 10 mL of sterile LB broth medium and homogenized. The experiment was conducted in triplicate, and the bacterial-inoculated LB broth medium was maintained for 48 h at 28 ± 2 °C on a 120 rpm rotary shaker [88]. The OD of the culture was determined at 600 nm using a UV-Vis spectrophotometer.

Similarly, the temperature tolerance of bacterial isolates was checked by preparing LB broth medium and sterilizing it at 121 °C for 15 min. About 10 µL of isolate culture was inoculated in 20 mL of LB broth and slightly vortexed [89]. The inoculated broth medium was maintained at 20, 30, 35, 40, and 45 °C for 48 h at 28 ± 2 °C. The experiment was conducted in triplicate. The OD of bacterial growth culture was measured at 600 nm in a UV-Vis spectrophotometer (Model: UV-1800, Shimadzu, Kyoto, Japan). As stated by Beal et al. [90], OD600 is the most used technique due to its speed, simplicity, non-destructive nature, and compatibility with high-throughput assays.

### 4.6. Molecular Characterization of Bacterial PGPR Strain

Isolates like So-1, So-12, and Ha-2 were selected for molecular identification because of their consistent performance across a range of PGP traits. These isolates exhibited at least three to four major PGP traits like IAA production, potassium solubilization, enzyme activity (cellulase, lipase, and amylase), and stress tolerance properties. These broad-spectrum in vitro findings revealed the isolates as potential bioinoculants, and they were further selected for 16S rRNA gene sequencing and phylogenetic analysis.

For molecular identification, bacterial genomic DNA was isolated with the Nucleospin microbial DNA kit (TAKARA) and used as a PCR template, with slight alterations [91]. For 16S rDNA amplification, we utilized the 16S rDNA bacterial universal primers, 27F (5′-AGAGTTTGATCMTGGCTCAG-3′) and 1492R (5′-TACGGYTACCTTGTTACGACTT-3′). The PCR analysis was performed in a 20 μL reaction mixture, which included 10 ng of genomic DNA as a template, 8 μL of PCR master mixer, 0.5 μL of a forward primer, and 0.5 μL of a reverse primer, and 20 μL was obtained by adding nuclease-free water to the final reaction mixture.

The PCR amplification was performed by employing a BigDye Terminator Cycle Sequencing Ready Reaction Kit (Applied Biosystems ABI3500 DNA Sequencer, Bengaluru, Karnataka, India). Afterwards, the purified products were analyzed through gel electrophoresis after purification by the commercially available purification kit. The purified DNA products were also outsourced to Greenarray Genomic Research & Solutions Private Limited, Pune, Maharashtra, for DNA sequencing. The 16S rDNA sequences obtained from the isolated PGPR strains So-1, So-12, and Ha-2 were submitted to the NCBI GenBank database, and accession numbers PQ432817.1, PQ432822.1, and PQ432823.1 were assigned (Table S5). Sequence alignment and evolutionary relationship analysis, compared to the most closely related bacterial sequences obtained from the National Center for Biotechnology (NCBI) database, were compared with MEGA XII using the neighbor-joining algorithm [92].

### 4.7. Preparation of Bacterial Inoculum

The screened PGPR isolates were inoculated in 250 mL of LB broth medium first and shaken in a rotating incubator at 150 rpm at 28 ± 2 °C for 48 h. After incubation, bacterial cells were centrifuged at 6000 rpm for 10 min to harvest them and resuspended in sterile saline to make a concentration of 2 × 10^8^ CFU/mL, which was then adjusted to OD 0.2 at 620 nm using a UV-Vis spectrophotometer (Model: UV-1800, Shimadzu, Japan), according to the procedure given by Abd El-Daim and Bashandy [93] and Kurabachew and Wydra [94]. The standardized suspension was used as the stock solution. From this stock, tenfold dilutions were performed using sterile distilled water to obtain two other concentrations: 10^6^ CFU/mL and 10^7^ CFU/mL, which were utilized for further purposes.

### 4.8. In Vitro Response of the Bacterial Isolates on the Growth of Tomato Seedlings

The effect of PGPR strains on the germination and early growth of tomato seeds (cv. Durga Abhinav F1) was assessed using the standard filter paper method pointed out by Chabbi et al. [91]. The tomato seeds were surface sterilized by submerging them in 70% ethanol for 4–5 min, followed by treatment with a H_2_O_2_ solution for 2–5 min. They were then rinsed three times with sterile distilled water and air-dried aseptically.

Bacterial suspensions were taken from 48 h old cultures and were standardized to 10^6^, 10^7^, and 10^8^ CFU/mL concentrations using sterile distilled water, as per the procedure described in Section 4.7. Seeds were incubated in these bacterial suspensions with 1% sterile CMC for 1 h to ensure the adhesion of bacteria to the seed surface. Seeds were dried under sterile air for 1–2 h after treatment. For germination tests, 30 tomato seeds per replicate were placed on two layers of sterile filter paper moistened with the corresponding bacterial suspension in 9 cm Petri dishes. For controls, seeds were treated with sterile distilled water with 1% CMC without bacteria. The experiment was conducted in triplicate. For maintaining moisture, 5 mL of sterile distilled water was added to each Petri dish every other day. All the dishes were incubated at 28 ± 2 °C on a 12 h light/12 h dark photoperiod for 7 days. Germination was checked daily, and the seed was regarded as germinated when the radicle length was more than half the length of the seed. The root and shoot lengths were taken at the end of the incubation. The percentage germination and SVI were determined using the following formulas:

Germination rate (%) = Number of germinated seeds/ Total number of treated seeds × 100

SVI= Germination % × mean seedling length (cm)

Relative SVI was calculated by comparing the treatment SVI to the control as Relative SVI (%) = (SVI treatment / SVI control) × 100

### 4.9. Preparation of Cocopeat-Based Bioformulation and Evaluation of In Vivo Pot Trial

#### 4.9.1. Preparation of Cocopeat-Based Bioformulation

The most effective PGPR concentration found from in vitro tomato seed germination assays was used to develop a cocopeat-based bioformulation adapted from the procedure developed by the Central Coir Research Institute, Coir Board, Kalavoor [95], with minor modifications (Figure S4). A volume of 200 mL of bacterial suspension was mixed aseptically with 1 kg of sterilized cocopeat (700 g cocopeat + 300 g banana peel) and 10 g of sterile CMC as an adhesive. The substrate was prepared in a weight ratio of 70:30:1 (cocopeat–banana peel–CMC). Despite this, the bacterial culture was maintained at room temperature for 24–48 h and subsequently shade-dried to below 20% moisture content.

#### 4.9.2. Soil Characteristics Used for Experiment

Soil employed in the in vivo pot trial was obtained from an agricultural field located in Hamirpur (village Markanda), Himachal Pradesh. The climate of this region is subtropical to temperate, with moderate to high annual rainfall and a wide cropping system. The texture of the indigenous soil is loamy to sandy loamy. The physico-chemical properties of the soil revealed pH 7.49, electric conductivity 0.19 dS/m, organic carbon 0.72%, available nitrogen 430 kg/ha (medium to high), available phosphorus 13.94 kg/ha, and available potassium 204.96 kg/ha.

Before its use in the pot experiments, bulk soil was air-dried and sterilized by autoclaving at 121 °C for 1 h on two consecutive days to eliminate indigenous microbial populations and ensure experimental uniformity.

#### 4.9.3. Response of PGPR Bioformulation in Tomato Plant

For in vivo seed germination evaluation, the surface-sterilized seeds of tomato (cv. Durga Abhinav F1) were inoculated with a bacterial suspension (10^7^ CFU/mL) for 1–2 h in aseptic conditions. Inoculated and uninoculated tomato seeds were then sown in plastic pots (20 cm diameter × 15 cm height). The pot contained a mixture of sterilized soil and cocopeat in a proportion of 3:1, w/w. Next, three seeds were sown per pot. Each treatment contained three replicates. Environmental conditions during the experiment were 18 °C to 27 °C temperature, 62% to 89% relative humidity, and a 7–8 h day photoperiod. At the time of seed sowing, 50 g of the respective PGPR bioformulation was applied to each pot to enhance seed germination. After this, the reapplication was maintained by applying a 20 g formulation at 7-day intervals until 40 days. After germination, only one healthy seedling per pot was maintained to ensure uniformity, and the other seedlings were carefully removed. Control pots received an equal volume of sterile, uninoculated cocopeat. The 45-day pot trial experiment was conducted using a fully randomized design under polyhouse conditions. After the end of the experiment, plant growth parameters such as root-shoot, fresh, and dry biomass were measured, following the protocol given by Mengistie and Awlachew [59], Almaghrabi et al. [55], and Egamberdieva et al. [96]. Additionally, agronomic parameters were analyzed using Pearson correlation coefficient analysis [39].

### 4.10. Chlorophyll Estimation

Apart from growth observations, biochemical parameters were also quantified to analyze the impact of PGPR bioformulation on plant performance. Chlorophyll content was estimated at 45 days after sowing using the method described by Meena et al. [97]. In this method, one gram of fresh fine leaf tissue was ground in a mortar using 20 mL of 80% acetone and centrifuged at 5000 rpm for 15 min at 4 °C. The residue was re-extracted with 80% acetone until colorless, and all supernatants were pooled in a 100 mL volumetric flask. The mortar was rinsed with acetone, and the final volume was made up to 100 mL. Absorbance was recorded at 645 nm and 663 nm using a UV–Vis spectrophotometer (Model: UV-1800, Shimadzu, Japan), and the total chlorophyll content was calculated and expressed as mg/g fresh weight. The following formula was used for calculating the chlorophyll level of the tomato leaves, according to the method given by Patani et al. [66]:

Chlorophyll ‘a’ (mg/g FW) = (12.7 × O.D. at 663 nm) − (2.69 × O.D. at 645 nm)

Chlorophyll ‘b’ (mg/mL FW) = (22.9 × O.D. at 645 nm) − (4.08 × O.D. at 663 nm)

Total chlorophyll (mg/mL FW) = (20.2 ×O.D. at 645 nm) + (8.02 × O.D. at 663 nm)

where FW indicates the fresh weight of the leaves.

### 4.11. Statistical Analysis

Data from in vitro experiments are given as mean ± SD of three replicates. One-way analysis of variance (ANOVA), along with the Bonferroni post hoc test, was performed to compare significant differences between isolates at *p* ≤ 0.05. In vivo inoculation experiments were carried out in a completely randomized design (CRD) and were analyzed by one- or two-way ANOVA to compare the effect of bacterial inoculation and other parameters, and significance was calculated at *p* ≤ 0.05. The relationship between agronomic parameters was assessed through Pearson’s correlation coefficient analysis.

## 5. Conclusions

This study provides a strong foundation for the use of indigenous PGPR strains *S. marcescens* So-1 and *Enterobacter* spp. So-12 and Ha-2 as potential bioinoculants in sustainable tomato cultivation. Based on the insights from the early-stage growth enhancements, future studies will assess the green area of the leaf, field trials, evaluation across a spectrum of tomato genotypes and solanaceous crops, and formulation development to achieve stable, scalable products. Follow-up research will have to employ advanced analytical methods like HPTLC or HPLC for the precise quantitation and profiling of indole compounds. Genomic analysis will be conducted to employ biosafety and test functional attributes, providing a new window into the introduction of these strains in eco-friendly agriculture systems.

## Figures and Tables

**Table 1:** Evaluation of growth-promoting, biocontrol potential, and antagonistic assay to determine the antifungal activity of rhizobacterial isolates.

Isolate Name	Growth-Promoting Activities		Biocontrol Activity	Antagonistic Effect of *F. oxysporum* (SR266-9)	
	Ammonia Production	EPS Production	HCN	Fungal Growth Diameter (cm)	Growth Inhibition (%)
Control	−	−	−	4.6	0
So-1	++++	+	+++	3.2	30.4 ± 0.85 ^a^
So-12	++++	+	+	−	−
So-21	−	+	+	−	−
Sr-17	++	+	−	3.1	32.6 ± 1.15 ^a^
Ha-1	+	−	−	−	−
Ha-2	+++	+	++	2.9	36.9 ± 1.00 ^b^
Un-7	+	−	−	−	−
Bl-2	++	−	++++	−	−
Bl-9	++	+	−	−	−
Ka-2	−	−	−	−	−

A “+” or “−” sign indicates the presence or absence of a PGP trait, and a measure of intensity is given by the number of “+” signs. Different lowercase letters within a column indicate significant variation between isolates using Bonferroni post hoc test at α = 0.05.

**Table 2:** PGPR treatment combinations and control used for in vitro and in vivo evaluation.

Treatment Code	PGPR Strain/Formulation	Description
**T1**	*Serratia marcescens* So-1	Single isolate
**T2**	*Enterobacter* sp. So-12	Single isolate
**T3**	*Enterobacter cloacae* Ha-2	Single isolate
**T4**	So-1 + So-12 + Ha-2	Consortium
**T5**	So-1 + So-12	Dual combination
**T6**	So-1 + Ha-2	Dual combination
**T7**	So-12 + Ha-2	Dual combination
**Control (C)**	---	Uninoculated control

**Table 3:** Pearson correlation coefficients for agronomic parameters.

	Shoot Length (cm)	Root Length (cm)	Fresh Weight of Shoot (g)	Fresh Weight of Root (g)	Dry Weight of Shoot (g)	Dry Weight of Root (g)
Shoot length (cm)	1					
Root length (cm)	0.633	1				
Fresh weight of shoot (g)	0.812	0.733	1			
Fresh weight of root (g)	0.621	0.855	0.797	1		
Dry weight of shoot (g)	0.920	0.796	0.893	0.744	1	
Dry weight of root (g)	0.784	0.874	0.908	0.793	0.924	1

## Data Availability

The original contributions presented in this study are included in the article/Supplementary Materials. Further inquiries can be directed to the corresponding authors.

## Supplementary Materials

The following supporting information can be downloaded at: https://www.mdpi.com/article/10.3390/plants14142154/s1, Figure S1: Biochemical characterization, plant growth promotion, biocontrol activity, and hydrolytic enzyme production; Figure S2: Linear standard curve of IAA concentrations (µg/mL) vs. their respective OD values; Figure S3: Preparation and evaluation of PGPR-based cocopeat bioformulation for in vivo testing; Figure S4: In vitro tomato seed germination observed on day 3 at a concentration of 10^7^ CFU/mL showed enhanced early growth and vigor over the control; Table S1: List of districts and corresponding locations in the Lower Western Himalayan region of Himachal Pradesh, India, from which rhizospheric soil samples of heathy tomato plants were collected; Table S2: Morphological, biochemical and PGP characteristics of the selected rhizobacteria isolated from the rhizosphere of tomato; Table S3: Evaluation of solubilization potential (K, IAA, and hydrolytic enzymes including lipase, cellulase, and amylase) of selected rhizobacterial isolates; Table S4: Selected rhizobacterial isolates were screened for their tolerance to a range of temperatures and pH conditions; Table S5: Species closely related to the bacterial species were identified by Blastn analysis of its 16S rRNA gene sequence; Table S6: Effect of different PGPR concentrations on in vitro seed germination and seedling growth parameters.

## Abbreviations

The following abbreviations are used in this manuscript:

PGPR	Plant Growth-Promoting Rhizobacteria
SVI	Seed Vigor Index
PGP	Plant Growth Promoting
CFU	Colony-Forming Unit
CMC	Carboxymethyl cellulose
IAA	Indole-3-Acetic Acid
SI	Solubilizing Index

## References

[1] Hasan A., Tabassum B., Hashim M., Khan N. (2024). Role of plant growth promoting rhizobacteria (PGPR) as a plant growth enhancer for sustainable agriculture: A review. Bacteria.

[2] Bhardwaj I., Kumar V., Bhardwaj N., Verma R., Bhardwaj Y., Kumari T. (2023). A glimpse into the performance and synthesis of microbial nanoparticles and its new advances in soil enrichment and plant nutrition: A review. Nanotechnol. Environ. Eng..

[3] Bhardwaj R., Yadav A., Sahoo A., Kumari P., Singh L.A., Swapnil P., Meena M., Kumar S. (2025). Microalgal-based sustainable bio-fungicides: A promising solution to enhance crop yield. Discov. Sustain..

[4] Goswami S.K., Singh V., Chakdar H., Choudhary P. (2018). Harmful effects of fungicides—Current status. Int. J. Agric. Environ. Biotechnol..

[5] Singh V.K., Meena M., Zehra A., Tiwari A., Dubey M.K., Upadhyay R.S. (2014). Fungal toxins and their impact on living systems. Microbial Diversity and Biotechnology in Food Security.

[6] Kürklü A., Pearson S., Felek T. (2025). Climate change impacts on tomato production in high-tech soilless greenhouses in Türkiye. BMC Plant Biol..

[7] Collins E.J., Bowyer C., Tsouza A., Chopra M. (2022). Tomatoes: An extensive review of the associated health impacts of tomatoes and factors that can affect their cultivation. Biology.

[8] Padmanabhan P., Cheema A., Paliyath G. (2016). Solanaceous fruits including tomato, eggplant, and peppers. Encyclopedia of Food and Health.

[9] Quinet M., Angosto T., Yuste-Lisbona F.J., Blanchard-Gros R., Bigot S., Martinez J.P., Lutts S. (2019). Tomato fruit development and metabolism. Front. Plant Sci..

[10] FAOSTAT. https://www.fao.org/faostat/en/#home.

[11] DesiKheti (2024). Top 10 Tomato-Producing States in India: A Detailed Statewise Overview. https://knowledge.desikheti.com/top-10-tomato-producing-states-in-india-a-detailed-statewise-overview/.

[12] District Solan, Government of Himachal Pradesh Mushroom City of India. https://hpsolan.nic.in/.

[13] Times of India (2022). Off-Season Tomatoes a Hit in HP, Plan to Hike Production. https://timesofindia.indiatimes.com/city/shimla/off-season-tomatoes-a-hit-in-hp-plan-to-hike-production/articleshow/92686994.cms.

[14] Kumar V., Sharma H., Sharma D. (2018). Profile and problems of tomato cultivation in Bilaspur district of Himachal Pradesh. Him. J. Agric. Res..

[15] National Horticulture Board (2018). Tomato—October 2018. https://nhb.gov.in/statistics/Reports/TomatoOctober2018.pdf.

[16] CEIC (2025). India Production of Horticulture Crops in Major States: Vegetables: Tomato. https://www.ceicdata.com/en/india/production-of-horticulture-crops-in-major-states-vegetables-tomato.

[17] Shubham, Rao A., Kaushal S. (2024). The Multifaceted Soils of Himachal Pradesh, India: Types and Characteristics. Arch. Curr. Res. Int..

[18] Panno S., Caruso A.G., Davino S., Bertacca S., Cravero V., Catara A., Garibaldi A., Minuto A. (2021). A review of the most common and economically important diseases that undermine the cultivation of tomato crop in the Mediterranean basin. Agronomy.

[19] Khlode R.S., Abdel-Gawad T.I., El-Bana A.A., Galal A.A. (2016). *Fusarium oxysporum* f. sp. *lycopersici* (fol) race 1 and 3 as wilt-incitants to tomato plants growing at El-Minia Governorate, Egypt. Minia J. Agric. Res. Dev..

[20] Gupta G., Parihar S.S., Ahirwar N.K., Snehi S.K., Singh V. (2015). Plant growth-promoting rhizobacteria (PGPR): Current and future prospects for development of sustainable agriculture. J. Microb. Biochem. Technol..

[21] Ma Y., Dias M.C., Freitas H. (2020). Drought and salinity stress responses and microbe-induced tolerance in plants. Front. Plant Sci..

[22] Aksoy A., Kaymak H.Ç. (2024). Tomato production quantity estimates for 2023–2027 with ARIMA model: Evidence from leading producing countries including Turkey. Sci. Pap. Ser. Manag. Econ. Eng. Agric. Rural Dev..

[23] Prasad R., Kumar M., Varma A., Egamberdieva D., Shrivastava S., Varma A. (2015). Role of PGPR in Soil Fertility and Plant Health. Plant-Growth-Promoting Rhizobacteria (PGPR) and Medicinal Plants.

[24] Fasusi O.A., Amoo A.E., Babalola O.O. (2021). Characterization of plant growth-promoting rhizobacterial isolates associated with food plants in South Africa. Antonie Van Leeuwenhoek.

[25] Fasusi O.A., Cruz C., Babalola O.O. (2021). Agricultural sustainability: Microbial biofertilizers in rhizosphere management. Agriculture.

[26] Gupta R., Solanki M.K., Saini S., Saxena A.K. (2022). Identification, characterization and optimization of phosphate-solubilizing rhizobacteria (PSRB) from rice rhizosphere. Saudi J. Biol. Sci..

[27] Khan N., Bano A., Babar M.A., Babar M. (2020). Water conservation and plant survival strategies of rhizobacteria under drought stress. Agronomy.

[28] Arora N.K., Tewari S., Singh R., Arora N. (2013). Multifaceted plant-associated microbes and their mechanisms diminish the concept of direct and indirect PGPRs. Plant Microbe Symbiosis: Fundamentals and Advances.

[29] Asari S., Tarkowská D., Rolčík J., Novák O., Palmero D., Bejai S., Meijer J. (2017). Analysis of plant growth-promoting properties of *Bacillus amyloliquefaciens* UCMB5113 using *Arabidopsis thaliana* as host plant. Planta.

[30] Ummara U., Noreen S., Afzal M., Ahmad P. (2021). Bacterial bioaugmentation enhances hydrocarbon degradation, plant colonization and gene expression in diesel-contaminated soil. Physiol. Plant..

[31] Odelade K.A., Babalola O.O. (2019). Bacteria, fungi and archaea domains in rhizospheric soil and their effects in enhancing agricultural productivity. Int. J. Environ. Res. Public Health.

[32] Dogan A., Aydogdu M., Ates F., Oguz A., Ilkme G., Yildirim H., Ozer G. (2018). Microbial-based production system: A novel approach for plant growth and pest and disease management in greenhouse-grown peppers (*Capsicum annuum* L.). J. Agric. Sci. Technol..

[33] Lori M., Symnaczik S., Mäder P., De Deyn G., Gattinger A. (2017). Organic farming enhances soil microbial abundance and activity—A meta-analysis and meta-regression. PLoS ONE.

[34] Chromkaew Y., Kaeomuangmoon T., Mawan N., Mukjang N., Khongdee N. (2023). Is coconut coir dust an efficient biofertilizer carrier for promoting coffee seedling growth and nutrient uptake?. PeerJ.

[35] de Andrade L.A., Santos C.H.B., Frezarin E.T., Sales L.R., Rigobelo E.C. (2023). Plant growth-promoting rhizobacteria for sustainable agricultural production. Microorganisms.

[36] Tang J., Li Y., Zhang L., Mu J., Jiang Y., Fu H., Zhang Y., Cui H., Yu X., Ye Z. (2023). Biosynthetic pathways and functions of indole-3-acetic acid in microorganisms. Microorganisms.

[37] Kaur N., Sharma P. (2013). Screening and characterization of native *Pseudomonas* sp. as plant growth-promoting rhizobacteria in chickpea (*Cicer arietinum* L.) rhizosphere. Afr. J. Microbiol. Res..

[38] Kalimuthu R., Suresh P., Varatharaju G., Balasubramanian N., Rajasekaran K.M., Shanmugaiah V. (2019). Isolation and characterization of indole acetic acid (IAA) producing tomato rhizobacterium *Pseudomonas* sp. VSMKU4050 and its potential for plant growth promotion. Int. J. Curr. Microbiol. Appl. Sci..

[39] Lata D.L., Abdie O., Rezene Y. (2024). IAA-producing bacteria from the rhizosphere of chickpea (*Cicer arietinum* L.): Isolation, characterization, and their effects on plant growth performance. Heliyon.

[40] Khoso M.A., Wagan S., Alam I., Hussain A., Ali Q., Saha S., Poudel T.R., Manghwar H., Liu F. (2024). Impact of plant growth-promoting rhizobacteria (PGPR) on plant nutrition and root characteristics: Current perspective. Plant Stress.

[41] Khan I., Mohyuddin S.G., Sohail Zaman S., Qadir M., Guo J., Li G. (2024). Enhancing growth in Vigna radiata through the inhibition of charcoal rot disease: A strategic approach using plant growth-promoting rhizobacteria. Microorganisms.

[42] Alonazi M.A., Alwathnani H.A., Al-Barakah F.N., Alotaibi F. (2025). Native plant growth-promoting rhizobacteria containing ACC deaminase promote plant growth and alleviate salinity and heat stress in maize (*Zea mays* L.) plants in Saudi Arabia. Plants.

[43] Azizah H., Rahajeng S.M., Jatmiko Y.D. (2020). Isolation and screening of phosphate and potassium solubilizing endophytic bacteria in maize (*Zea mays* L.). J. Exp. Life Sci..

[44] Zhao Y., Liang H., Zhang J., Chen Y., Dhital Y.P., Zhao T., Wang Z. (2024). Isolation and characterization of potassium-solubilizing rhizobacteria (KSR) promoting cotton growth in saline–sodic regions. Microorganisms.

[45] Abd El-Rahman A.F., Shaheen H.A., Abd El-Aziz R.M., Ibrahim D.S. (2019). Influence of hydrogen cyanide-producing rhizobacteria in controlling the crown gall and root-knot nematode, *Meloidogyne incognita*. Egypt. J. Biol. Pest Control.

[46] Mekonnen H., Kibret M., Assefa F. (2022). Plant growth promoting rhizobacteria for biocontrol of tomato bacterial wilt caused by *Ralstonia solanacearum*. Int. J. Agron..

[47] Yao X., Lan X., Jin Y., Li C. (2024). Screening, identification, and characterization of plant growth-promoting rhizobacterium strains from alpine grassland as biocontrol agents against *Fusarium oxysporum*. Agronomy.

[48] Singh R.P., Jha P.N. (2016). The multifarious PGPR *Serratia marcescens* CDP-13 augments induced systemic resistance and enhanced salinity tolerance of wheat (*Triticum aestivum* L.). PLoS ONE.

[49] Patel S.K., Singh S., Benjamin J.C., Singh V.R., Bisht D., Lal R.K. (2024). Plant growth-promoting activities of *Serratia marcescens* and *Pseudomonas fluorescens* on *Capsicum annuum* L. plants. Ecol. Front..

[50] Chakraborty U., Chakraborty B.N., Chakraborty A.P. (2010). Influence of *Serratia marcescens* TRS-1 on growth promotion and induction of resistance in *Camellia sinensis* against *Fomes lamaoensis*. J. Plant Interact..

[51] Zaheer A., Mirza B.S., Mclean J.E., Yasmin S., Shah T.M., Malik K.A., Mirza M.S. (2016). Association of plant growth-promoting *Serratia* spp. with the root nodules of chickpea. Res. Microbiol..

[52] Abreo E., Altier N. (2019). Pangenome of *Serratia marcescens* strains from nosocomial and environmental origins reveals different populations and the links between them. Sci. Rep..

[53] Sharma A., Chakdar H., Vaishnav A., Srivastava A.K., Khan N., Bansal Y.K., Kaushik R. (2023). Multifarious plant growth-promoting rhizobacterium *Enterobacter* sp. CM94-mediated systemic tolerance and growth promotion of chickpea (*Cicer arietinum* L.) under salinity stress. Front. Biosci.-Landmark.

[54] Zhang X., Peng J., Hao X., Feng G., Shen Y., Wang G., Chen Z. (2024). *Serratia marcescens* LYGN1 reforms the rhizosphere microbial community and promotes cucumber and pepper growth in plug seedling cultivation. Plants.

[55] Almaghrabi O.A., Massoud S.I., Abdelmoneim T.S. (2013). Influence of inoculation with plant growth promoting rhizobacteria (PGPR) on tomato plant growth and nematode reproduction under greenhouse conditions. Saudi J. Biol. Sci..

[56] Zhao Y., Hong Y., Wang P., Gou Y., Zeng R., Zhang Q., Chen D., Song Y. (2023). Assembly of tomato rhizobacteria from different functional groups improves seedling photosynthesis and growth. Plants.

[57] Fiodor A., Ajijah N., Dziewit L., Pranaw K. (2023). Biopriming of seed with plant growth-promoting bacteria for improved germination and seedling growth. Front. Microbiol..

[58] González-Ista N.S., Castro-Mercado E., de la Cruz H.R., Campos-García J., López-Bucio J., García-Pineda E. (2023). Comparison of the rhizobacteria *Serratia* sp. H6 and *Enterobacter* sp. L7 on *Arabidopsis thaliana* growth promotion. Curr. Microbiol..

[59] Mengistie G.Y., Awlachew Z.T. (2022). Evaluation of the plant growth promotion effect of *Bacillus* species on different varieties of tomato (*Solanum lycopersicum* L.) seedlings. Adv. Agric..

[60] Singh A., Yadav V.K., Chundawat R.S., Soltane R., Awwad N.S., Ibrahium H.A., Yadav K.K., Vicas S.I. (2023). Enhancing plant growth promoting rhizobacterial activities through consortium exposure: A review. Front. Bioeng. Biotechnol..

[61] Rosier A., Beauregard P.B., Bais H.P. (2021). Quorum quenching activity of the PGPR *Bacillus subtilis* UD1022 alters nodulation efficiency of *Sinorhizobium meliloti* on *Medicago truncatula*. Front. Microbiol..

[62] Abou Jaoudé R., Luziatelli F., Ficca A.G., Ruzzi M. (2025). Effect of plant growth-promoting rhizobacteria synthetic consortium on growth, yield, and metabolic profile of lettuce (*Lactuca sativa* L.) grown under suboptimal nutrient regime. Horticulturae.

[63] Mekonnen H., Kibret M., Assefa F., Kabtimer N. (2025). Potential of plant growth-promoting rhizobacteria for enhancement of tomato growth. Agrosyst. Geosci. Environ..

[64] He S., Li L., Lv M., Wang R., Wang L., Yu S., Gao Z., Li X. (2024). PGPR: Key to enhancing crop productivity and achieving sustainable agriculture. Curr. Microbiol..

[65] Myresiotis C.K., Karaoglanidis G.S., Vryzas Z., Papadopoulou-Mourkidou E. (2012). Evaluation of plant-growth-promoting rhizobacteria, acibenzolar-S-methyl and hymexazol for integrated control of *Fusarium* crown and root rot on tomato. Pest Manag. Sci..

[66] Patani A., Prajapati D., Ali D., Kalasariya H., Yadav V.K., Tank J., Bagatharia S., Joshi M., Patel A. (2023). Evaluation of the growth-inducing efficacy of various *Bacillus* species on the salt-stressed tomato (*Lycopersicon esculentum* Mill.). Front. Plant Sci..

[67] Dhole S.M., Amnerkar N.D., Khedekar P.B. (2012). Comparison of UV spectrophotometry and high performance liquid chromatography methods for the determination of repaglinide in tablets. Pharm. Methods.

[68] Hegyi A., Nguyen T.B.K., Posta K. (2021). Metagenomic analysis of bacterial communities in agricultural soils from Vietnam with special attention to phosphate solubilizing bacteria. Microorganisms.

[69] Brar B., Kumar R., Sharma D., Sharma A.K., Thakur K., Mahajan D., Kumar R. (2023). Metagenomic analysis reveals diverse microbial community and potential functional roles in Baner rivulet, India. J. Genet. Eng. Biotechnol..

[70] Qingwei Z., Lushi T., Yu Z., Yu S., Wanting W., Jiangchuan W., Xiaolei D., Xuejiao H., Bilal M. (2023). Isolation and characterization of phosphate-solubilizing bacteria from rhizosphere of poplar on road verge and their antagonistic potential against various phytopathogens. BMC Microbiol..

[71] Stewart E.J. (2012). Growing unculturable bacteria. J. Bacteriol..

[72] Sharma R.C., Dogra S. (2011). Characterization of the soils of lower Himalayas of Himachal Pradesh, India. Nat. Environ. Pollut. Technol..

[73] Mengesha A.S., Legesse N.H. (2024). Isolation and characterization of phosphate solubilizing bacteria from the rhizosphere of lentil (*Lens culinaris* M.) collected from Hagere Mariam district, Central Ethiopia. PLoS ONE.

[74] Arjyal C., Kc J., Neupane S. (2020). Prevalence of Methicillin-Resistant *Staphylococcus aureus* in Shrines. Int. J. Microbiol..

[75] Radhakrishnan N., Krishnasamy C. (2024). Isolation and characterization of salt-stress-tolerant rhizosphere soil bacteria and their effects on plant growth-promoting properties. Sci. Rep..

[76] Hiranmayee G., Marik D., Sadhukhan A., Reddy G.S. (2023). Isolation of plant growth-promoting rhizobacteria from the agricultural fields of Tattiannaram, Telangana. J. Genet. Eng. Biotechnol..

[77] Pallavi, Mishra R.K., Sahu P.K., Mishra V., Jamal H., Varma A., Tripathi S. (2023). Isolation and characterization of halotolerant plant growth promoting rhizobacteria from mangrove region of Sundarbans, India for enhanced crop productivity. Front. Plant Sci..

[78] Sherpa M.T., Lepcha S.R., Rai P., Arora D.K. (2021). Isolation and characterization of plant growth promoting rhizobacteria isolated from organically grown high yielding pole type native pea (*Pisum sativum* L.) variety Dentami of Sikkim, India. Curr. Res. Microb. Sci..

[79] Nawaz A., Chaudhary H.J., Zhang H., Mukhtar H., Hussain S., Siddique M.H., Haseeb M., Mehmood K., Muneer M.A., Ismail M. (2023). Contribution of potassium solubilizing bacteria in improved potassium assimilation and cytosolic K^+^/Na^+^ ratio in rice (*Oryza sativa* L.) under saline-sodic conditions. Front. Microbiol..

[80] Alotaibi F., St-Arnaud M., Hijri M. (2022). In-depth characterization of plant growth promotion potentials of selected alkanes-degrading plant growth-promoting bacterial isolates. Front. Microbiol..

[81] Shreshtha K., Rai B., Sharma R., Pradhan A. (2025). Isolation and characterization of plant growth promoting rhizobacteria from cacti root under drought condition. Curr. Res. Microb. Sci..

[82] Vasant G., Bhatt S., Raghav R. (2023). Isolation and molecular characterization of plant growth promoting rhizobacteria from groundnut (*Arachis hypogaea* L.) rhizosphere. Curr. Agric. Res. J..

[83] Tsegaye Z., Woldesenbet F., Mulaw T., Sahile S. (2019). Isolation and biochemical characterization of plant growth promoting (PGP) bacteria colonizing the rhizosphere of Tef crop during the seedling stage. J. Plant Sci. Phytopathol..

[84] Yasmin H., Naz R., Nosheen A., Hassan M.N., Ilyas N., Sajjad M., Anjum S., Gao X., Geng Z. (2020). Identification of new biocontrol agent against charcoal rot disease caused by *Macrophomina phaseolina* in soybean (*Glycine max* L.). Sustainability.

[85] Raklami A., Bechtaoui N., Tahiri A.I., Anli M., Meddich A., Oufdou K. (2019). Use of rhizobacteria and mycorrhizae consortium in the open field as a strategy for improving crop nutrition, productivity and soil fertility. Front. Microbiol..

[86] Ganesh J., Hewitt K., Devkota A.R., Wilson T., Kaundal A. (2024). IAA-producing plant growth-promoting rhizobacteria from *Ceanothus velutinus* enhance cutting propagation efficiency and *Arabidopsis* biomass. Front. Plant Sci..

[87] Agarwal M., Dheeman S., Dubey R.C., Kumar P., Maheshwari D.K., Bajpai V.K. (2017). Differential antagonistic responses of *Bacillus pumilus* MSUA3 against *Rhizoctonia solani* and *Fusarium oxysporum* causing fungal diseases in *Fagopyrum esculentum* Moench. Microbiol. Res..

[88] Divyanshu K., Kumari A., Dixit S., Singh S.K., Yadav A.K., Pandey V., Srivastava M. (2022). Molecular identification and characterization of plant growth-promoting rhizobacteria and their effect on seed germination and vigour index of barley (*Hordeum vulgare* L.). J. Pure Appl. Microbiol..

[89] Ndeddy Aka R.J., Babalola O.O. (2016). Effect of bacterial inoculation of strains of *Pseudomonas aeruginosa*, *Alcaligenes faecalis* and *Bacillus subtilis* on germination, growth and heavy metal (Cd, Cr, and Ni) uptake of *Brassica juncea*. Int. J. Phytoremediation.

[90] Beal J., Farny N.G., Haddock-Angelli T., Selvarajah V., Baldwin G.S., Buckley-Taylor R., Gershater M., Kiga D., Marken J., Sanchania V. (2020). Robust estimation of bacterial cell count from optical density. Commun. Biol..

[91] Chabbi N., El Harchli E.H., El Yamani M., Rahou Y., Rahou A., El Farissi M., Ouhammou A. (2024). Plant-growth-promoting rhizobacteria improve seeds germination and growth of *Argania spinosa*. Plants.

[92] Thakur R., Dhar H., Swarnkar M.K., Soni R., Sharma K.C., Singh A.K., Gulati A., Sud R.K., Gulati A. (2024). Understanding the molecular mechanism of PGPR strain *Priestia megaterium* from tea rhizosphere for stress alleviation and crop growth enhancement. Plant Stress.

[93] Abd El F.E.Z.A., Bashandy S.R. (2019). Dose-dependent effects of *Pseudomonas trivialis* rhizobacteria and synergistic growth stimulation effect with earthworms on the common radish. Rhizosphere.

[94] Kurabachew H., Wydra K. (2013). Characterization of plant growth promoting rhizobacteria and their potential as bioprotectant against tomato bacterial wilt caused by *Ralstonia solanacearum*. Biol. Control.

[95] CSIR-North East Institute of Science and Technology (2018). Development of Commercial Bioformulation of PGPR Using Coir Pith as a Carrier.

[96] Egamberdieva D., Davranov K., Wirth S., Hashem A., Abd_Allah E.F. (2017). Impact of soil salinity on the plant-growth–promoting and biological control abilities of root associated bacteria. Saudi J. Biol. Sci..

[97] Meena P., Rai A.K. (2017). Biochemical analysis of PGPR and its effect on chlorophyll, ascorbic acid, starch & total polyphenolic content (TPC) of different varieties of wheat (*Triticum aestivum*). Pharma Innov..

