# Supplementary material for: Evaluation of Stress-Tolerant Serratia and Enterobacter as PGPR for Nutrient Solubilization and Dose-Dependent Bioformulation to Enhance Tomato Seedlings
Source: Plants (Basel). 2025 Jul 13;14(14):2154. doi: 10.3390/plants14142154 (PMC12299828; doi:10.3390/plants14142154)
Supplement: Supplementary file 1 [file plants-14-02154-s001.zip › plants-3725026-supplementary.pdf]

District name	Locations						Number of samples collected In each district
	Site 1	Site 2	Site 3	Site 4	Site 5	Site 6	
Hamirpur	Markanda	Bhoranj	Barsar	Awah Devi	Ukhali	Sujanpur	6
Bilaspur	Ghumarwin	Kalri	Baddu	Dangar	Bharari	Panol	6
Una	Haroli	Amb	Dohak	Sanoli	Mehatpur	Behdala	6
Solan	Nauni	Dharo	Basal	Kandaghat	Jaunaji	Oachghat	6
Sirmour	Bharoli	Jol	Dharoti	Badiana	Manal	Rajgarh	6
Kangra	Nurpur	Dehra	Jawali	Haripur	Nagrota Surian	Fatehpur	6

Supplementary Table S1. List of districts and corresponding locations in the Lower Western Himalayan region of Himachal Pradesh, India, from which rhizospheric soil samples of healthy tomato plants were collected.

S. No.	Isolates name	Morphological features of the isolate		Biochemical tests						
		Gram reaction	Shape	Oxidase	Mannitol fermentation	Catalase	Citrate	Urease	TSI	Methyl Red
**1.**	**So-1**	-ve	Rod	+	+	+	+	+	-	+
**2.**	**So-12**	+ve	Rod	+	+	+	+	-	+	+
**3.**	**So-21**	-ve	Rod	-	-	+	+	+	-	+
**4.**	**Sr-17**	+ve	Bacilli	+	-	+	+	-	-	-
**5.**	**Ha-1**	-ve	Rod	+	-	+	-	-	-	-
**6.**	**Ha-2**	-ve	Rod	+	-	+	+	+	-	+
**7.**	**Un-7**	+ve	Bacilli	+	-	-	-	-	-	-
**8.**	**Bl-2**	-ve	Bacilli	+	-	+	+	-	-	+
**9.**	**Bl-9**	-ve	Rod	+	+	+	+	-	-	+
**10.**	**Ka-2**	-ve	Rod	+	+	-	+	-	-	-

Supplementary Table S2. Morphological, biochemical, and PGP characteristics of the selected rhizobacteria isolated from the rhizosphere of tomato plants

*Legend: + Positive result; – Negative result

Characterization of PGPR based on their PGP traits					
**Isolate name**	**Growth-promoting activities**	**Hydrolytic enzyme production activity**			**Phyto-hormone production activity**
	**K-solubilization index (KSI)**	**Lipase SI**	**Amylase SI**	**Cellulase SI**	**IAA**
**So-1**	2.38 ± 0.78[a]	1.56 ± 0.05[a]	1.59 ± 0.08[a]	-	24.1 ± 0.02[a]
**So-12**	-	1.47 ± 0.06[ac]	1.28 ± 0.06[b]	1.72 ± 0.07[a]	20.9 ± 0.02[b]
**So-21**	-	1.78 ± 0.08[b]	1.59 ± 0.03[a]	2.20 ± 0.08[b]	13.2 ± 0.01[c]
**Sr-17**	-	1.26 ± 0.07[c]	-	1.59 ± 0.08[a]	2.6 ± 0.01[d]
**Ha-1**	-	2.20 ± 0.08[d]	1.58 ± 0.04[a]	3.19 ± 0.05[c]	3.8 ± 0.01[e]
**Ha-2**	2.25 ± 0.53[a]	1.31 ± 0.06[c]	-	2.39 ± 0.09[b]	14.3 ± 0.01[f]
**Un-7**	-	1.66 ± 0.09[ab]	-	-	2.7 ± 0.01[g]
**Bl-2**	1.61 ± 0.19[a]	1.22 ± 0.06[c]	-	-	4.3 ± 0.02[h]
**Bl-9**	-	1.69 ± 0.07[ab]	1.71 ± 0.05[a]	1.58 ± 0.02[a]	2.6 ± 0.02[d]
**Ka-2**	5.73 ± 0.30[b]	-	-	-	6.4 ± 0.01[i]

Supplementary Table S3. Evaluation of the solubilization potential of selected rhizobacterial isolates. Data represents the means ± SD of at least three replicates. Statistical significance was assessed using ANOVA at 5 % significance level (P value < 0.05). Different lowercase letters within a column indicate significant variation between isolates using the Bonferroni post-hoc test at $\alpha = 0.05$.

Isolates	Temperature					pH		
	20 ºC	30 ºC	35 ºC	40 ºC	45 ºC	3	7	9
**So-1**	0.30 ± 0.03[a]	0.95 ± 0.02[a]	1.09 ± 0.02[a]	0.51 ± 0.04[a]	0.12 ± 0.02[a]	0.71 ± 0.02[a]	1.09 ± 0.03[a]	0.24 ± 0.03[a]
**So-12**	0.24 ± 0.03[b]	0.63 ± 0.02[b]	0.83 ± 0.02[b]	0.44 ± 0.03[a]	0.08 ± 0.01[a]	0.43 ± 0.02[b]	0.80 ± 0.02[b]	0.19 ± 0.03[ac]
**So-21**	0.05 ± 0.02[c]	0.21 ± 0.02[c]	0.64 ± 0.03[c]	0.15 ± 0.03[b]	0.02 ± 0.01[b]	0.14 ± 0.02[c]	0.54 ± 0.03[c]	0.03 ± 0.01[b]
**Sr-17**	0.12 ± 0.02[d]	0.16 ± 0.01[c]	0.25 ± 0.02[d]	0.08 ± 0.01[b]	0.06 ± 0.03[a]	0.26 ± 0.03[d]	0.39 ± 0.03[d]	0.02 ± 0.01[b]
**Ha-1**	0.02 ± 0.02[c]	0.4 ± 0.03[d]	0.58 ± 0.03[c]	0.19 ± 0.02[b]	0.07 ± 0.02[a]	0.31 ± 0.01[d]	0.48 ± 0.01[c]	0.13 ± 0.02[c]
**Ha-2**	0.13 ± 0.02[d]	0.55 ± 0.02[e]	1.03 ± 0.02[a]	0.33 ± 0.03[c]	0.06 ± 0.02[a]	0.39 ± 0.02[b]	0.70 ± 0.02[e]	0.21 ± 0.04[a]
**Un-7**	0.01 ± 0.01[c]	0.23 ± 0.02[c]	0.54 ± 0.04[c]	0.17 ± 0.02[b]	0.07 ± 0.02[a]	0.39 ± 0.02[b]	0.56 ± 0.04[c]	0.05 ± 0.01[b]
**Bl-2**	0.23 ± 0.02[b]	0.59 ± 0.02[e]	0.65 ± 0.04[c]	0.17 ± 0.02[b]	0.03 ± 0.01[a]	0.03 ± 0.01[e]	0.64 ± 0.02[e]	0.04 ± 0.02[b]
**Bl-9**	0.05 ± 0.01[c]	0.33 ± 0.02[f]	0.51 ± 0.02[c]	0.07 ± 0.01[b]	0.03 ± 0.02[a]	0.24 ± 0.02[d]	0.40 ± 0.02[d]	0.15 ± 0.03[c]
**Ka-2**	0.03 ± 0.02[c]	0.49 ± 0.02[e]	0.67 ± 0.05[c]	0.13 ± 0.02[b]	0.02 ± 0.02[b]	0.13 ± 0.01[c]	0.67 ± 0.04[e]	0.19 ± 0.03[ac]

Supplementary Table S4. Selected rhizobacterial isolates were screened for their tolerance to various temperatures and pH conditions. Data represents the means ± SD of at least three replicates. Statistical significance was assessed using ANOVA at 5 % significance level (P value < 0.05). Different lowercase letters within a column indicate significant variation between isolates using the Bonferroni post-hoc test at α = 0.05.

PGPR strain	Accession no.	GenBank database		
		Top-hit strain	% identity	Accession no.
So-1	PQ432817.1	*Serratia marcescens*	99.22 %	MH396732.1
So-12	PQ432822.1	*Enterobacter sp.*	98.66 %	OP990266.1
Ha-2	PQ432823.1	*Enterobacter cloacae* subsp. dissolvens	98.99 %	MK789855.1

Supplementary Table S5. Species closely related to the bacterial species were identified by Blastn analysis of their 16S rRNA gene sequence

Evaluation of in vitro tomato seed germination and seed vigor index on day 7							
**Treatment**	**Concentration**	**Total seeds in each Petri plate**	**% seed germination**	**Root length (cm)**	**Shoot length (cm)**	**SVI**	**Relative SVI (%)**
**T1** (So-1)		30	80.0 ± 1.00^{a}	4.90 ± 0.20^{a}	2.73 ± 0.12^{a}	610.4	315.6
**T2** (So-12)		30	75.6 ± 1.53^{b}	3.93 ± 0.15^{b}	2.50 ± 0.17^{a}	486.4	251.4
**T3** (Ha-2)		30	70 ± 1.00^{c}	4.07 ± 0.15^{b}	2.17 ± 0.15^{a}	436.3	225.6
**T4** (T1+T2+T3)	10^{6}	30	59 ± 1.53^{d}	2.83 ± 0.25^{d}	2.07 ± 0.15^{a}	289.1	149.5
**T5** (T1+T2)		30	63.3 ± 1.00^{e}	3.63 ± 0.15^{b}	1.80 ± 0.30^{ab}	343.9	177.8
**T6)** (T1+T3)		30	46.3 ± 1.15^{f}	3.37 ± 0.15^{b}	2.30 ± 0.26^{a}	262.4	135.7
**T7** **(**T2+T3)		30	46.6 ± 1.00^{f}	3.83 ± 0.15^{b}	2.07 ± 0.15^{a}	274.9	142.1
**T8** Control	Distilled water with 1% CMC	30	44.3 ± 1.53^{f}	2.93 ± 0.15^{d}	1.43 ± 0.25^{b}	193.4	100
**T1** (So-1)		30	92.3 ± 0.58^{a}	5.47 ± 0.15^{a}	2.47 ± 0.29^{a}	732.2	378.6
**T2** (So-12)		30	83.3 ± 1.00^{b}	4.50 ± 0.20^{be}	2.70 ± 0.10^{a}	599.8	310.1
**T3** (Ha-2)		30	82.3 ± 0.58^{b}	3.97 ± 0.12^{b}	2.90 ± 0.20^{a}	565.1	292.3
**T4** (T1+T2+T3)		30	52.3 ± 0.58^{d}	3.33 ± 0.21^{d}	2.23 ± 0.12^{a}	291.1	150.5
**T5** (T1+T2)	10^{7}	30	70 ± 1.00^{e}	4.60 ± 0.20^{e}	1.93 ± 0.06^{a}	457.3	236.5
**T6)** (T1+T3)		30	41 ± 1.15^{f}	3.40 ± 0.20^{d}	2.47 ± 0.32^{a}	240.5	124.4
**T7** **(**T2+T3)		30	82.3 ± 1.53^{b}	4.97 ± 0.21^{ae}	1.67 ± 0.15^{b}	545.9	282.2
**T8** Control	Distilled water with 1% CMC	30	44.3 ± 1.53^{c}	2.93 ± 0.15^{d}	1.43 ± 0.25^{b}	193.4	100
**T1** (So-1)		30	82.3 ± 0.58^{a}	4.03 ± 0.21^{a}	2.23 ± 0.15^{a}	515.7	266.5
**T2** (So-12)		30	76.6 ± 1.00^{b}	4.13 ± 0.21^{a}	2.67 ± 0.12^{a}	520.9	269.5

**T3** (Ha-2)	10^8	30	71 ± 0.58^{c}	3.53 ± 0.21^{a}	2.53 ± 0.21^{a}	430.7	222.6
**T4** (T1+T2+T3)		30	61 ± 0.58^{d}	2.13 ± 0.21^{b}	2.77 ± 0.15^{a}	298.9	154.5
**T5** (T1+T2)		30	57.6 ± 1.53^{d}	3.67 ± 0.15^{a}	1.43 ± 0.15^{b}	293.8	151.9
**T6)** (T1+T3)		30	44.3 ± 1.53^{e}	2.87 ± 0.21^{c}	2.40 ± 0.17^{a}	233.3	120.6
**T7** **(**T2+T3)		30	64.3 ± 1.53^{d}	3.40 ± 0.26^{ac}	2.53 ± 0.21^{a}	381.5	197.3
**T8** Control	Distilled water with 1% CMC	30	44.3 ± 1.53^{e}	2.93 ± 0.15^{c}	1.43 ± 0.25^{b}	193.4	100

Supplementary Table S6. Effect of different PGPR concentrations on in vitro seed germination and seedling growth parameters. Each concentration group included its control (uninoculated seeds), and comparisons were made within each concentration level only, between the control and the respective PGPR treatments. Data represents the means ± SD of at least three replicates. Statistical significance was assessed using ANOVA at 5 % significance level (P value < 0.05). Different lowercase letters within a column indicate significant variation between isolates using the Bonferroni post-hoc test at α = 0.05.

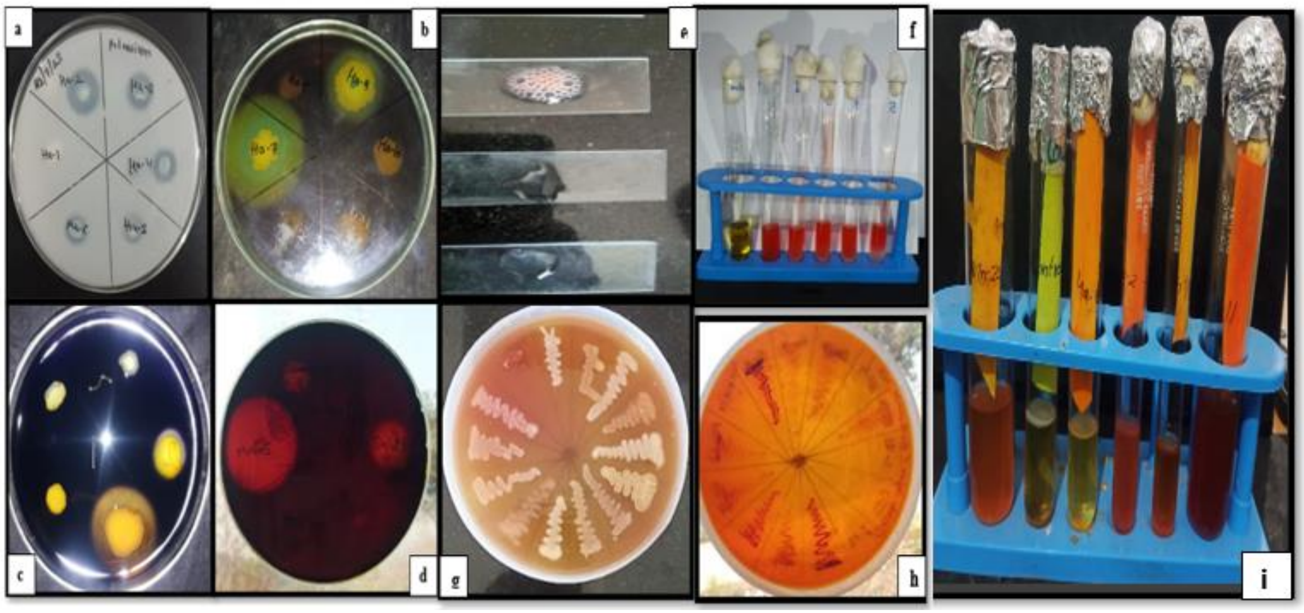

Supplementary Figure S1. shows biochemical characterization, plant growth promotion, biocontrol activity, and hydrolytic enzyme production **(a)** K-solubilization; **(b)** lipase; **(c)** amylase; **(d)** cellulase activity; **(e)** catalase biochemical test; **(f)** methyl red test; **(g)** mannitol fermentation test; **(h)** citrate agar test; and **(i)** HCN production.

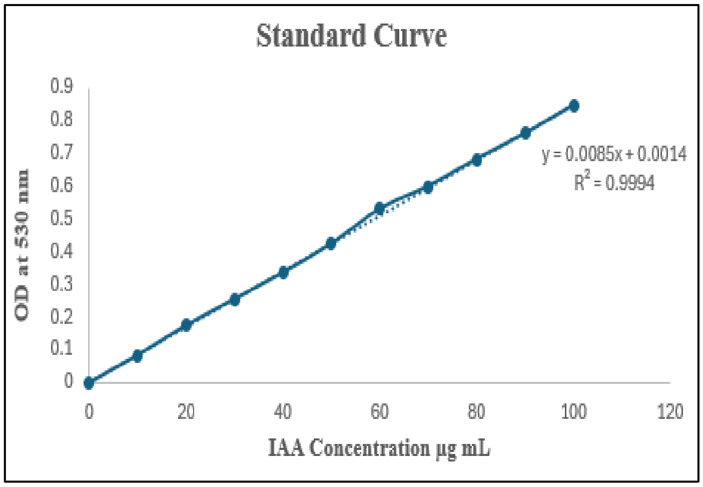

Supplementary Figure S2 shows a Linear standard curve of IAA concentrations (µg/mL) vs their respective OD values.

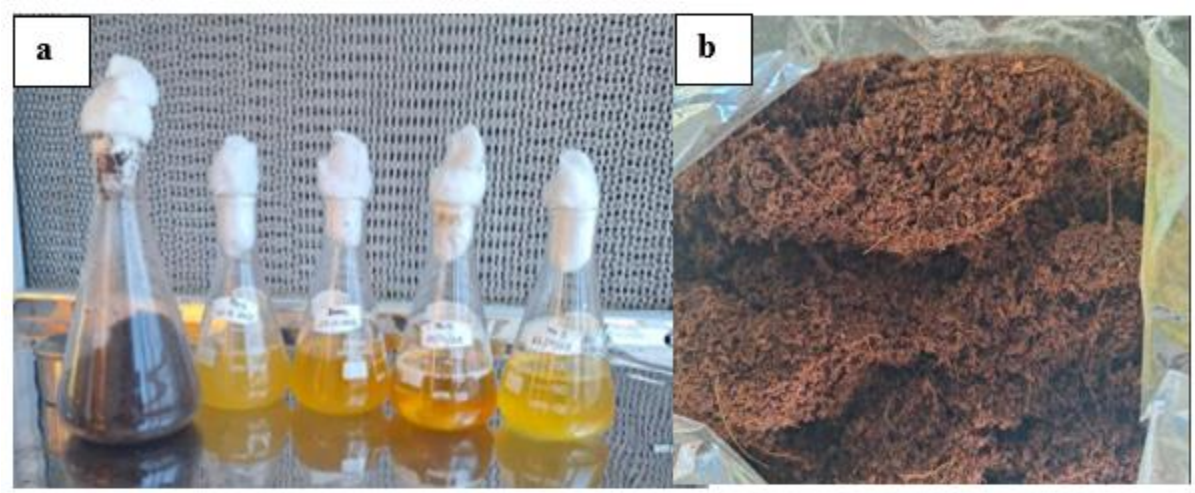

Supplementary Figure S3. (a-b) Preparation and evaluation of PGPR-based cocopeat bioformulation for in vivo testing

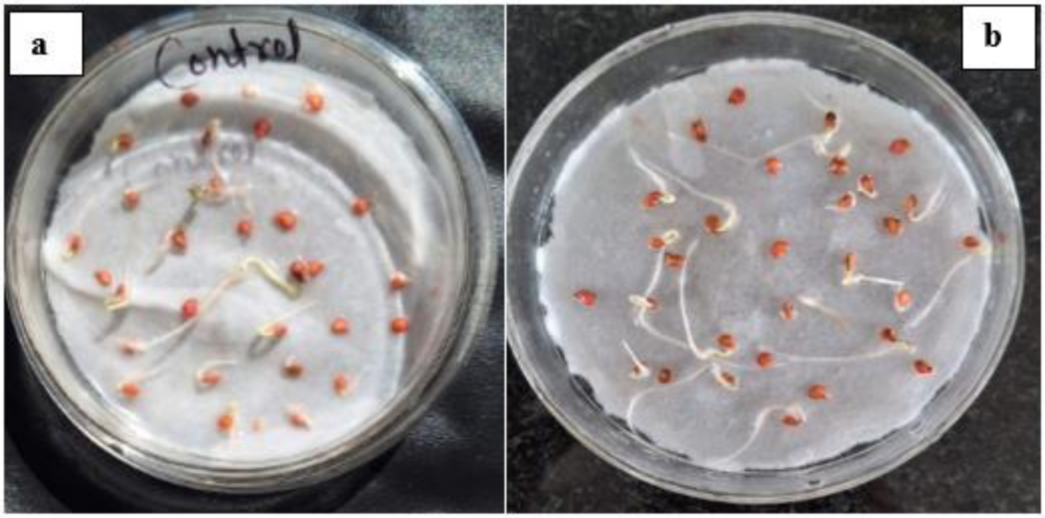

Supplementary Figure S4. (a) Control T8; and **(b)** T1 show in vitro tomato seed germination observed on day 3 at a concentration of 10^7 CFU/mL, showing enhanced early growth and vigor
